# Analysis of Antibodies to Newly Described Plasmodium falciparum Merozoite Antigens Supports MSPDBL2 as a Predicted Target of Naturally Acquired Immunity

**DOI:** 10.1128/IAI.00301-13

**Published:** 2013-10

**Authors:** Kevin K. A. Tetteh, Faith H. A. Osier, Ali Salanti, Gathoni Kamuyu, Laura Drought, Marilyne Failly, Christophe Martin, Kevin Marsh, David J. Conway

**Affiliations:** Department of Pathogen Molecular Biology, London School of Hygiene and Tropical Medicine, London, United Kingdoma; Pathogen Vector and Human Biology Department, KEMRI-Centre for Geographical Medicine Research, Coast, Kilifi, Kenyab; Centre for Medical Parasitology, Institute of International Health, Immunology and Microbiology, Copenhagen, Denmarkc; PX Therapeutics, Minatec, Grenoble, Franced

## Abstract

Prospective studies continue to identify malaria parasite genes with particular patterns of polymorphism which indicate they may be under immune selection, and the encoded proteins require investigation. Sixteen new recombinant protein reagents were designed to characterize three such polymorphic proteins expressed in Plasmodium falciparum schizonts and merozoites: MSPDBL1 (also termed MSP3.4) and MSPDBL2 (MSP3.8), which possess Duffy binding-like (DBL) domains, and SURFIN4.2, encoded by a member of the surface-associated interspersed (*surf*) multigene family. After testing the antigenicities of these reagents by murine immunization and parasite immunofluorescence, we analyzed naturally acquired antibody responses to the antigens in two cohorts in coastal Kenya in which the parasite was endemic (Chonyi [*n* = 497] and Ngerenya [*n* = 461]). As expected, the prevalence and levels of serum antibodies increased with age. We then investigated correlations with subsequent risk of clinical malaria among children <11 years of age during 6 months follow-up surveillance. Antibodies to the polymorphic central region of MSPDBL2 were associated with reduced risk of malaria in both cohorts, with statistical significance remaining for the 3D7 allelic type after adjustment for individuals' ages in years and antibody reactivity to whole-schizont extract (Chonyi, risk ratio, 0.51, and 95% confidence interval [CI], 0.28 to 0.93; Ngerenya, risk ratio, 0.38, and 95% CI, 0.18 to 0.82). For the MSPDBL1 Palo Alto allelic-type antigen, there was a protective association in one cohort (Ngerenya, risk ratio, 0.53, and 95% CI, 0.32 to 0.89), whereas the other antigens showed no protective associations after adjustment. These findings support the prediction that antibodies to the polymorphic region of MSPDBL2 contribute to protective immunity.

## INTRODUCTION

An effective malaria vaccine is needed, particularly against Plasmodium falciparum, which causes most disease and mortality. Trials of the lead preerythrocytic stage candidate vaccine—RTS, S/ASO2—have shown partial protection of short duration, suggesting that addition of antigens of the blood stage may be needed to achieve higher levels of efficacy (1). Evidence suggests that such a vaccine would need to incorporate important target antigens on the surface of the invasive merozoite or the infected erythrocyte (2). Characterization of naturally acquired human antibody responses to specific antigens has been undertaken to describe associations with protection from clinical malaria, highlighting a need for simultaneous analysis of multiple antigens (3–5). Analysis of transcripts and proteins (6–9) and genomic (10, 11) and population genetic (12–16) studies of P. falciparum have identified new genes that may encode promising candidates for a vaccine.

High-throughput short-read sequencing of P. falciparum-infected blood samples in populations where they are endemic has recently allowed population genetic studies to shift from studying candidate molecules to screen most of the protein-coding genes in the parasite genome (17–19). Here, we investigate immune responses to protein products of three genes expressed at the merozoite stage that showed evidence of balancing selection in a genome-wide scan of a Gambian population (18), with similar results when tested separately in a Kenyan population (16). They are MSPDBL1 (also referred to as MSP3.4 [20]; gene locus PF3D7_1035700, previously PF10_0348) and MSPDBL2 (also referred to as MSP3.8 [20]; gene locus PF3D7_1036300, previously PF10_0355), which are members of the MSP3 family possessing a central Duffy-binding-like (DBL) region, and SURFIN4.2 (a member of the *surf* gene family; locus PF3D7_0424400, previously PFD1160w) (16, 21, 22). Recent studies have indicated a role for both MSPDBL1 and MSPDBL2 in binding to the erythrocyte surface (23, 24), with the interaction mediated by the DBL region (24). The gene encoding MSPDBL2 showed the strongest evidence of balancing selection in each of the previous studies (16, 18), and gene knockout or episomal overexpression affects parasite growth in the presence of some drugs *in vitro* (25, 26).

In this study, 16 new recombinant proteins based on polymorphic and conserved parts of these antigens were designed and expressed. Each of the antigens elicited murine antibodies reactive with P. falciparum schizonts and was then assayed for reactivity with naturally acquired antibodies in cohorts of individuals living in two villages in coastal Kenya where the parasite is endemic. Antibodies against one allelic form of MSPDBL2 were significantly associated with protection from malaria in both cohorts, even after adjusting for potential confounding variables, such as age and exposure, while only one other recombinant antigen showed a protective association in one cohort and the remaining 14 in neither cohort.

## MATERIALS AND METHODS

### Ethics statement.

Ethical approval for the study on samples from human subjects was obtained from the Kenya National Research Ethics Committee, the University of Oxford, and the London School of Hygiene and Tropical Medicine. Written informed consent was obtained from a parent or guardian of each child contributing a blood sample and also from participating adults. Murine antibodies were obtained commercially by immunization of mice under commercial subcontract, and all animal work protocols were approved and licensed by the United Kingdom Home Office as governed by law under the Animals (Scientific Procedures) Act of 1986, in strict accordance with the Code of Practice Part 1 for the housing and care of animals (21 March 2005), available at http://www.homeoffice.gov.uk/science-research/animal-research/.

### Cloning and expression of recombinant antigens in E. coli and baculovirus systems.

**Fig 1:**
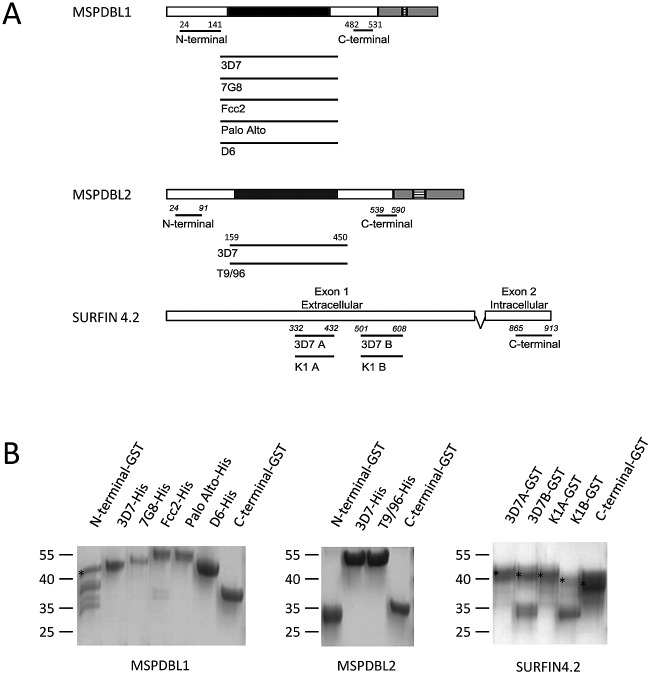
Sixteen new recombinant proteins representing different sequences within the P. falciparum merozoite antigens MSPDBL1, MSPDBL2, and SURFIN4.2. (A) Scheme of the antigens showing, by horizontal bars below each antigen, the positions (amino acid numbering according to the 3D7 reference sequence) and different allelic types of the sequences expressed. Black shading indicates DBL domains. Gray shading represents Surface Protein Associated with Merozoites (SPAM) domains common to the MSP3-like antigen family (hatching represents repeat sequences within the SPAM domain). The SURFIN4.2 sequences, along with N- and C-terminal regions of other antigens, were expressed in E. coli as GST fusion proteins. The central polymorphic regions of both MSPDBL1 and MSPDBL2 were expressed in baculovirus as 6×His-tagged proteins. (B) Coomassie-stained 4 to 20% gradient SDS-PAGE showing E. coli-expressed GST-tagged proteins and baculovirus-expressed His-tagged proteins. Including fusion tags, the expected product sizes of the recombinant antigens listed from left to right are as follows: MSPDBL1 products, 38, 41, 41, 41, 41, 41, and 32 kDa; MSPDBL2 products, 34, 41, 41, and 31 kDa; SURFIN4.2 products, 37, 38, 38, 39, and 32 kDa. For products with additional bands, presumably caused by proteolysis during production, the band closest to the size of the expected complete product is indicated with an asterisk.

Sixteen new constructs were designed (Fig. 1A); 9 smaller fragments without predicted disulfide bonds were expressed in Escherichia coli, and 7 larger fragments with predicted intramolecular disulfide bonds were expressed in baculovirus.

### E. coli-expressed GST-tagged fusion proteins.

Sequences in the N- and C-terminal regions encoded by the P. falciparum genes *mspdbl1* (PF3D7_1035700, previously PF10_0348; nucleotide positions 97 to 414 and 1441 to 1590 based on the 3D7 reference sequence) and *mspdbl2* (PF3D7_1036300, previously PF10_0355; nucleotides [nt] 70 to 273 and 1615 to 1770) and a C-terminal intracellular sequence encoded by exon 2 of *surf_4.2_* (PF3D7_0424400, previously PFD1160w; nucleotides 2593 to 2739) were PCR amplified from regions of each gene showing minimal polymorphism (see Figure S1 in the supplemental material). Four allelic constructs (representing the divergent 3D7 and K1 alleles) were PCR amplified from a polymorphic extracellular sequence encoded within exon 1 of *surf_4.2_* (PF3D7_0424400; nt 994 to 1296 and 1501 to 1824) (see Figure S1 in the supplemental material). DNA for each construct was PCR amplified from 3D7 genomic DNA (and K1 for *surf_4.2_*), cloned into the pGEM-T Easy TA vector (Promega), and sequence verified. Correct sequence inserts were subcloned into the pGEX-2T expression vector (GE Healthcare), sequenced again to ensure fidelity, and transformed into E. coli BL21(DE3) cells for expression. Expression and affinity purification were performed as described previously for other glutathione *S*-transferase (GST) fusion proteins (27).

### Baculovirus-expressed His-tagged fusion proteins.

Five polymorphic antigens based on the *mspdbl1* gene (PF3D7_1035700, previously PF10_0348) and two polymorphic antigens based on the *mspdbl2* gene (PF3D7_1036300, previously PF10_0355), covering the central DBL region, were expressed using a baculovirus expression system (28). PCR primers amplified a region within *mspdbl1* and *mspdbl2* (nt 418 to 1323 and 475 to 1350, respectively, based on the 3D7 genome sequence; accession number AAN35552) (Fig. 1A; see Figure S1 in the supplemental material). The amplified products were cloned into a pGEM-T Easy TA vector and sequence verified for generation of baculovirus constructs following procedures described for other DBL-containing antigens (28). Briefly, correct sequences were subcloned into the pAcGP67-A (BD Biosciences) baculovirus vector modified to contain a V5 epitope upstream of a C-terminal His tag. Recombinant virus was generated by cotransfecting the modified pAcGP67-A vector into Sf9 insect cells with linearized Bakpak6 Baculovirus DNA (BD Biosciences). The transfected Sf9 cells were then used to infect a High-Five cell suspension grown in a serum-free medium (Gibco). Recombinant protein was harvested from culture supernatant 26 h after viral infection, 0.2-μm filtered, and dialyzed into buffer (500 mM NaCl, 10 mM NaH_2_PO_4_, pH 7.4) using an Akta cross-flow (GE Healthcare).

### Generation of polyclonal sera in mice and immunofluorescence assays (IFA).

Groups of 5 CD1 outbred mice were immunized with 25 μg of each of the 16 recombinant antigens emulsified in Freund's complete adjuvant following a 60-day protocol (Pharmidex, United Kingdom), and boosting immunizations were performed twice more at 28-day intervals in Freund's incomplete adjuvant. Sera were collected before immunization and on days 14 and 42, and final serum collection was 7 days after the last immunization.

Antibody reactivities of murine antisera were tested against cultured P. falciparum 3D7 parasites using IFA. Parasite cultures with a large proportion of schizonts were washed in phosphate-buffered saline (PBS)-1% bovine serum albumin (BSA) and resuspended to 2.5% hematocrit, and 15-μl aliquots were spotted onto multiwell slides (Hendley, Essex, United Kingdom), which were then air dried and stored at −40°C with desiccant until required. Following a recommended fixation protocol (29), the slides were bathed in 4% paraformaldehyde in PBS for 30 min, followed by 10 min in 0.1% Triton X-100 in PBS, and then overnight at 4°C in PBS-3% BSA. After air drying, the wells were incubated with defined dilutions of each test serum (including initial serial doubling dilutions from 1/200 to 1/409,600) in PBS-3% BSA and incubated for 30 min at room temperature. The slides were rinsed 3 times in PBS, excess wash buffer was removed, and the wells were incubated for 30 min with a 1/500 dilution of biotinylated anti-mouse IgG (Vector Laboratories, USA) in PBS-3% BSA, washed 3 times in PBS, and incubated for 30 min with a 1/500 dilution of fluorescein-streptavidin (Vector Laboratories). Mounting medium with DAPI (4′,6-diamidino-2-phenylindole) (Vectashield; Vector Laboratories) was added to each slide and sealed with a coverslip for microscopy.

### Community surveys and human serum antibody analysis.

A community cohort study was undertaken in Chonyi and Ngerenya, two villages approximately 40 km apart in Kilifi district near the coast in eastern Kenya, samples from which have been previously analyzed for antibody responses to other malaria antigens (4, 30). At the time of sampling, Chonyi village had a higher endemicity rate than Ngerenya village (31). Inhabitants of these villages (predominantly of the Mijikenda ethnic group) were naturally exposed to biannual peaks of malaria transmission in November to December and May to July (with the latter generally being the most intense period of transmission). Blood samples were collected from individuals living in each location in October 2000, with the ages of sampled individuals ranging from 7 weeks to 85 years in Chonyi and from 3 weeks to 85 years in Ngerenya. The two cohorts were monitored by field workers weekly, with active and passive case detection conducted over 28 weeks, which included the lower of the two annual peaks of malaria transmission (November to January). A malaria episode was defined as a febrile episode (axillary temperature, >37.5°C), together with P. falciparum parasitemia of greater than 2,500 parasites μl blood^−1^, as determined by microscopic examination of thick blood smears, except for infants under 1 year of age, for whom any P. falciparum parasitemia plus fever was counted as malaria. This has been shown to comprise an accurate measure for malaria case detection in these populations (31).

Indirect enzyme-linked immunosorbent assays (ELISAs) were performed with each of the 16 antigens using protocols similar to those previously described for other merozoite antigens (4), with each serum sample tested in duplicate at 1/500 dilution. Sera were scored as positive for a particular antibody specificity if ELISA optical density (OD) values were higher than the mean plus 3 standard deviations of the values from 20 malaria-naive control sera tested in parallel (the same panel of negative-control sera was used in all assays). To test for cross-reactive epitopes on different antigens, and for the presence of heat-stable and heat-labile epitopes, competition ELISAs were performed using adult sera for which sufficient volumes were available, as was previously done for reactivity to other antigens (12). Tests for associations between antibody reactivities and occurrence of clinical malaria focused on individuals who were <11 years old and asymptomatically positive for P. falciparum by slide examination in October 2000, as this age group produced most of the subsequent clinical episodes and parasite-negative individuals include many with very low exposure to malaria (4, 32). Antibody reactivity to cultured parasite schizont extract was previously assayed in each of the sera, alongside analysis of other antigens (4, 30).

### Statistical analyses.

All analysis was performed with Stata/IC 11.2 (StataCorp LP, USA). Generalized linear models were used to determine the associated risk ratio (RR) between the presence or absence of detectable serum antibodies (IgG above the cutoff OD value) and occurrence of subsequent clinical malaria episodes. Individuals' ages in years and antibody reactivity to cultured parasite schizont extract were used in multivariate analyses to adjust for the confounding effects of variation among individuals in previous exposure to malaria.

## RESULTS

### Expression of new recombinant antigens.

To investigate naturally acquired immune responses to newly described P. falciparum antigens, 16 recombinant proteins based on polymorphic and conserved sequences were designed (Fig. 1). The amino acid positions of each of the recombinant proteins in relation to the 3D7 reference sequence are shown in Fig. 1A. From previous sequence analysis of diverse laboratory isolates and isolates from Kenya (16), allelic sequences of *mspdbl2* and *surf_4.2_* had both been shown to cluster into dimorphic allelic groups, so two divergent alleles for each of these genes were selected for expression. Two different regions that showed high levels of polymorphism were expressed from *surf_4.2_* (Fig. 1A). Allelic sequences of the *mspdbl1* gene also clustered into dimorphic types, but there was considerable subtype variation within the DBL region (15, 16), so five divergent allelic sequences were expressed to provide broader coverage of the diversity (see Figure S1 in the supplemental material). The central DBL domain region of the 5 chosen allelic sequences of *mspdbl1* (3D7, 7G8, Fcc2, Palo Alto, and D6) and two of *mspdbl2* (3D7 and T9/96) were baculovirus expressed, whereas all other antigens were expressed as GST-tagged fusion proteins in E. coli. All 16 recombinant antigens were assessed for size and purity by SDS-PAGE (Fig. 1B).

### Expressed recombinant antigens contain native epitopes.

Polyclonal sera to each antigen were raised by immunization of mice and tested for reactivity to native parasite schizonts by IFA. Parasite-specific antibodies elicited to each of the E. coli-expressed and baculovirus-expressed antigens were detected (see Figure S2 in the supplemental material). We previously noted that only a small proportion of mature schizont stage parasites were positive by IFA for the MSPDBL2 antigen (immature stage parasites were negative), using sera raised to the conserved N- and C-terminal antigens (18), and here, we also saw a minority reacting with antibodies to the antigen, whereas the antibodies raised to each of the other antigens generally reacted with all schizonts. No parasite-specific staining was observed with sera from nonimmunized mice.

### High prevalence of human antibodies to polymorphic and conserved antigens.

**Fig 2:**
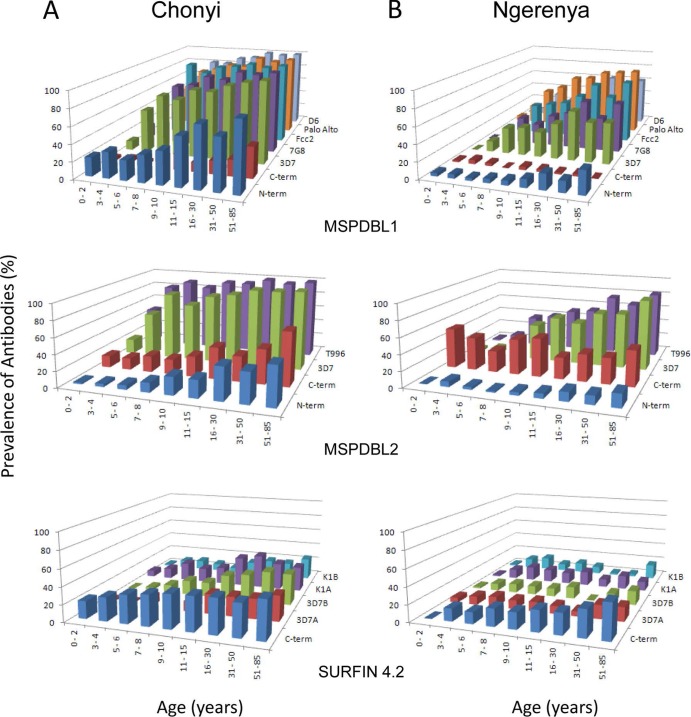
Age prevalences of naturally acquired serum IgG antibodies to the MSPDBL1, MSPDBL2, and SURFIN4.2 antigens in two Kenyan populations, Chonyi (high transmission; *n* = 497) (A) and Ngerenya (low transmission; *n* = 461) (B). Antibody positivity to each antigen was defined as ELISA reactivity above the mean plus 3 standard deviations of a panel of European negative-control sera as defined in Materials and Methods.

**Fig 3:**
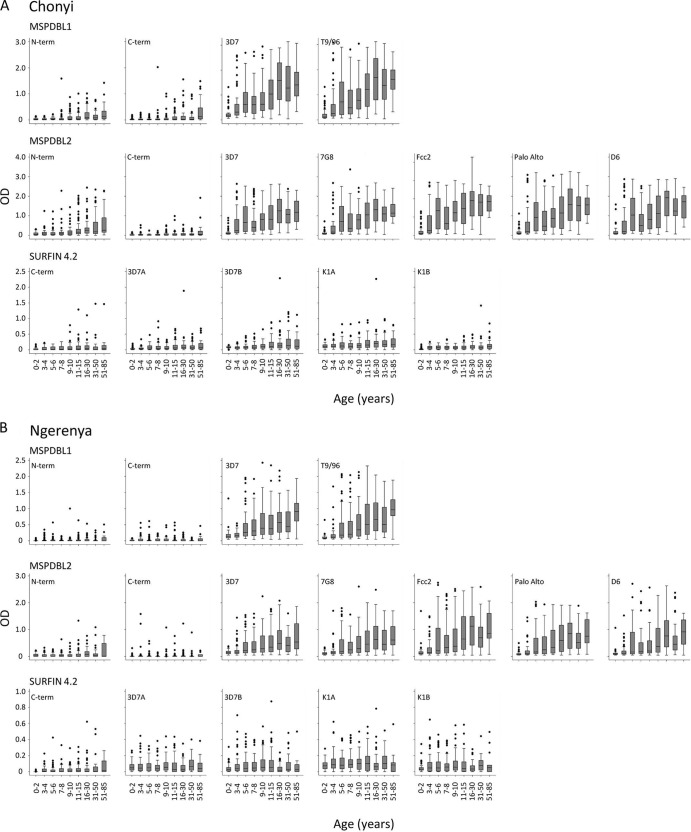
Age distribution of ELISA OD values for serum IgG against each of the antigens in Chonyi (A) and Ngerenya (B) villages. The medians are shown by the horizontal bars, and the boxes show the interquartile ranges (the whiskers denote upper and lower 95% CI limits).

Serum IgG antibody reactivity to all antigens was studied in all age groups in two coastal Kenyan rural populations, Chonyi (*n* = 497) and Ngerenya (*n* = 461). Antibody prevalence and OD levels were higher in Chonyi (high transmission) than in Ngerenya (low transmission) for most antigens tested. There was high antibody prevalence against polymorphic parts of MSPDBL1 and MSPDBL2, increasing in young children and rapidly approaching 100% in young adults (Fig. 2), while ELISA OD values also showed a steady increase with age into adulthood (Fig. 3). Antibody reactivity to the reagents representing conserved parts of these antigens was lower, as might be expected from relatively short recombinant antigen sequences (Fig. 2 and 3). Antibody prevalence and ELISA OD values against the expressed sequences of SURFIN4.2 were relatively low, with an increase with age against most of them apparent in Chonyi, but not in Ngerenya (Fig. 2 and 3).

Pearson's correlation analysis of ELISA OD values for reactivity to each of the recombinant antigens indicates the presence of both allele-specific and cross-reactive epitopes (see Table S1 in the supplemental material). In addition, high correlation coefficients were observed in comparison of reactivity profiles between MSPDBL1 and MSPDBL2, whereas cross correlations between either of them and the SURFIN4.2 antigens were much lower (see Table S1 in the supplemental material). Competition ELISAs using sera from two adult donors with high levels of antibodies were performed to test for cross-reactivity between antigens. Although most antibodies in these sera were specific for each separate antigen, with reactivity against allele-specific as well as conserved epitopes, some antibodies were cross-reactive between MSPDBL1 and MSPDBL2, and heat denaturation of competing antigens confirmed that some reactivity was against conformational epitopes (see Fig. S3 in the supplemental material).

### Antibodies associated with a reduced risk of malaria.

**Fig 4:**
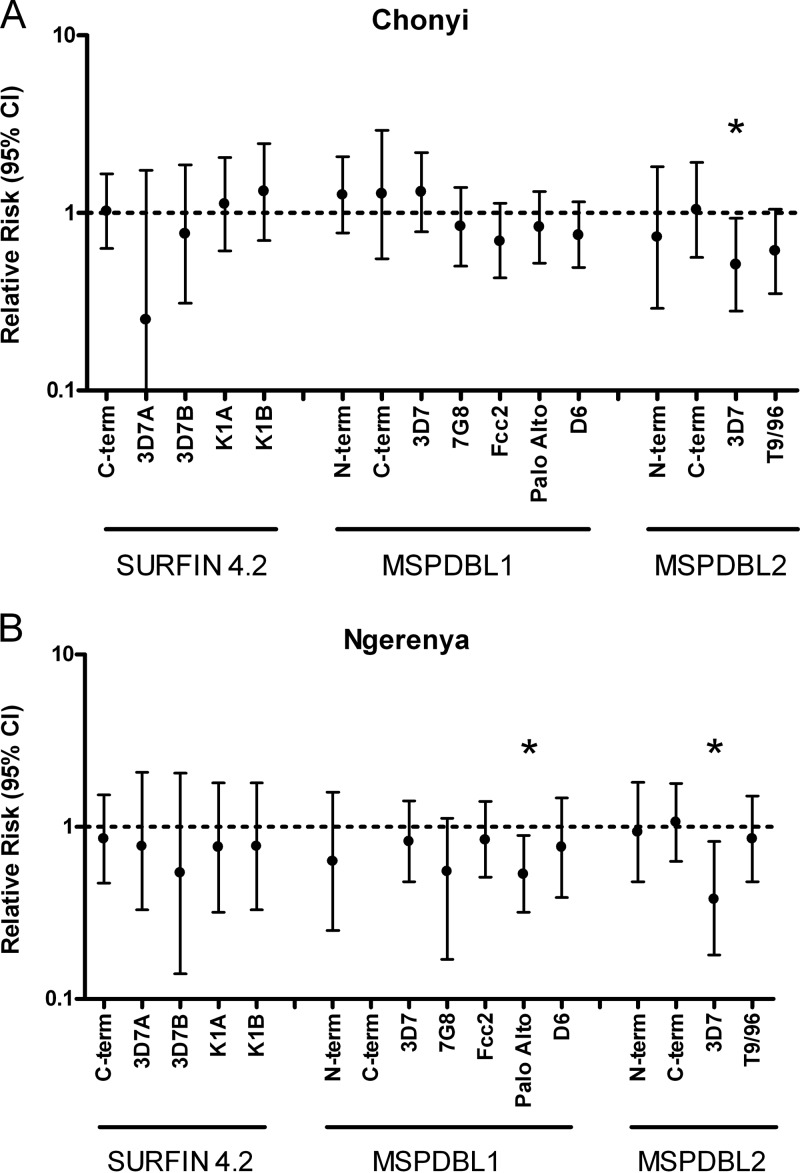
Two cohort studies yielded relative risk estimates (with 95% confidence intervals) of associations between antibody reactivity against each of a panel of 16 recombinant antigens at one time point and experience of clinical malaria during 6 months of follow-up. Analyses were conducted on data from children <11 years of age at the time of sampling for sera (October 2000) and adjusted for individuals' ages in years and reactivity to whole parasite schizont extract by logistic regression. The results are plotted separately for each cohort, Chonyi (A) and Ngerenya (B). Exact numbers from the analyses are given in Tables S2 and S3 in the supplemental material.

Tests for associations between serum IgG antibody reactivities and occurrence of clinical malaria during follow-up over the following 28 weeks focused on individuals who were <11 years old and asymptomatically positive for P. falciparum by slide examination at the time of serum collection in October 2000 (Chonyi, *n* = 119; Ngerenya, *n* = 61), following an approach taken in a previous study (4). This group was analyzed because >80% of all clinical episodes in these cohorts occurred in children <11 years old, and slide-positive children are likely to have recent or current exposure to malaria parasites, whereas among the parasite-negative individuals, it is not possible to differentiate those who are simply less exposed to malaria (4, 30, 33). Likely confounding effects of general differences in exposure were taken into account by multiple logistic regression analysis, adjusting for age and serum IgG reactivity to whole P. falciparum schizont extract. Analyses were tabulated separately for the Chonyi cohort (see Table S2 in the supplemental material) and the Ngerenya cohort (see Table S3 in the supplemental material), and the adjusted relative risk estimates are summarized in Fig. 4. In Ngerenya, individuals positive for antibodies to the Palo Alto allelic type of MSPDBL1 were less likely to develop malaria within the following 6 months (RR, 0.53; 95% confidence interval [CI], 0.32 to 0.89; *P* < 0.05), but this association was not significant in Chonyi. More significantly, antibodies to the 3D7 allelic type of DBLMSP2 were associated with a reduced subsequent risk of malaria in each cohort (Chonyi, RR, 0.51, 95% CI, 0.28 to 0.93, and *P* < 0.05; Ngerenya, RR, 0.38, 95% CI, 0.18 to 0.82, and *P* < 0.05) (Fig. 4; see Tables S2 and S3 in the supplemental material). For the remaining 14 antigens, antibodies were not associated with protection.

## DISCUSSION

This study involved the design of antigenic reagents based on recently described proteins encoded by three P. falciparum genes expressed at the schizont and merozoite stages that show evidence of being under balancing selection in populations where the parasite is endemic. A similar approach has been used to identify regions of more intensively studied candidate antigens (12, 27, 33) to guide vaccine design that may be based on either multiallelic formulation (34–39) or selective elicitation of responses to conserved epitopes (38, 40).

Here, human serum IgG prevalence was highest to the polymorphic regions of MSPDBL1 and MSPDBL2 in both of the Kenyan cohorts studied. Consistent with the fact that MSPDBL2 showed the strongest indication of balancing selection in a genome-wide analysis of P. falciparum (18), we observed that those with antibodies against one of the forms of this antigen had a lower risk of contracting malaria during the subsequent follow-up over 6 months. This result was independently significant in each of the cohorts, even after adjustment for individuals' ages in years and antibody reactivity to whole parasite schizont extract. It is unclear why the protective association was seen with only one of the major allelic forms, as both major forms of MSPDBL2 were common in Ngerenya when sampled shortly before the cohort study was conducted (16). These results indicate that the MSPDBL2 antigen is likely to be an important target of immunity, although the highly divergent sequences of the two major allelic types (16) suggest a multiallelic formulation would be needed if the antigen were to be incorporated into a vaccine. Evidence that expression of MSPDBL2 varies among parasites (18, 25) also indicates that a vaccine could not be based on this antigen alone.

Comparisons of serological reactivity profiles demonstrated high correlations between the DBL regions of the MSPDBL1 and MSPDBL2 antigens, reported to be important for erythrocyte binding (23, 24), and the existence of cross-reactive epitopes on these proteins was confirmed by competition ELISAs with selected sera. Amino acid sequence alignment analysis of the DBL regions from both antigens revealed short sequences conserved between them (see Fig. S1C in the supplemental material). It is notable that the gene encoding MSPDBL1 has a second copy in a minority of P. falciparum lines (15), showing regions of identity with MSPDBL2, with a sequence of 10 amino acids (aa) identical at MSPDBL1 aa 183 to 192 and MSPDBL2 aa 199 to 208 and a sequence of 16 amino acids identical for 15 residues at MSPDBL1 aa 325 to 340 and MSPDBL2 aa 343 to 358, as identified by amino acid sequence alignment (41, 42), although we have not tested if these are epitope sequences. Although the overall predicted amino acid sequence identity among members of the MSP3 family is low, short stretches of sequence identity exist near the C terminus, and cross-reactive antibodies have been described (20) and investigated as potential vaccine targets (43). However, these previously described cross-reactive sequences were downstream of the DBL region and were not included in the antigenic constructs of MSPDBL1 and MSPDBL2 designed and analyzed here.

Studies such as these of antibody reactivity to recombinant proteins, even when associated with epidemiological outcomes, do not directly demonstrate a mechanism of immunity. It was previously shown (43) that each member of the MSP3 family (including MSPDBL1 and MSPDBL2) had a distinct IgG isotype profile, although the cytophilic subclasses IgG1 and IgG3 were dominant against each (40). Antibody-dependent cellular inhibition (ADCI) by monocytes against parasites in culture utilizes these subclasses and has been shown to involve antibodies against MSP3. Such a mechanism might be similarly effective against both MSPDBL1 and MSPDBL2, although this has not yet been tested.

Clearly, the appropriate selection of antigens is an essential step in the design and development of vaccines. Here, we have described the generation of a new panel of antigen reagents as part of a process to identify potential candidates that are targets of naturally acquired immunity. For one of these antigens, based on the 3D7 allelic type of the DBL region of MSPDBL2, antibodies were associated with reduced prospective risk of malaria in two different populations where the parasite is endemic. This is already a higher level of reproducibility than normally shown in studies of other candidate antigens (3), although studies on immune responses to the antigen in other populations are recommended to further evaluate its importance as a target of immunity.

## Supplementary Material

Supplemental material
