# Supplementary material for: Analysis of Antibodies to Newly Described Plasmodium falciparum Merozoite Antigens Supports MSPDBL2 as a Predicted Target of Naturally Acquired Immunity
Source: Infect Immun. 2013 Oct;81(10):3835–42. doi: 10.1128/IAI.00301-13 (PMC3811751; doi:10.1128/IAI.00301-13)
Supplement: Supplemental material [file supp_81_10_3835__index.html]

Supplemental material 

# Analysis of Antibodies to Newly Described Plasmodium falciparum Merozoite Antigens Supports MSPDBL2 as a Predicted Target of Naturally Acquired Immunity

## Supplemental material

**Files in this Data Supplement:**

- Supplemental file 1 -

  Fig. S1. Amino acid sequence alignments. Fig. S2. Examples of parasites stained in an immunofluorescence assay with murine sera raised to each of the 16 recombinant antigens. Fig. S3. Competition ELISAs to test for the presence of cross-reactive, conformational, and linear epitopes in recombinant antigens. Table S1. Tabulated Pearson correlations from pairwise analysis for the full panel of 16 antigens for Chonyi and Ngerenya. Table S2. Association between the presence of serum IgG and the panel of 16 antigens in children aged <11 years and parasite slide positive in October 2000 in Chonyi village and the occurrence of an episode of clinical malaria over the following 6 months. Table S3. Association between the presence of serum IgG and the panel of 16 antigens in children aged <11 years and parasite slide positive in October 2000 in Ngerenya village and the occurrence of an episode of clinical malaria over the following 6 months.

  PDF, 1.7M
