# Supplementary material for: Analysis of Antibodies to Newly Described Plasmodium falciparum Merozoite Antigens Supports MSPDBL2 as a Predicted Target of Naturally Acquired Immunity
Source: Infect Immun. 2013 Oct;81(10):3835–42. doi: 10.1128/IAI.00301-13 (PMC3811751; doi:10.1128/IAI.00301-13)
Supplement: Supplemental material [file IAI.00301-13_zii999090337so1.pdf]

Supplementary Material

This file contains 3 supplementary figures (Figures S1-S3) and 3 supplementary tables (Tables S1-S3).

Supplementary Figure S1. (A) Amino acid sequence alignment highlighting the level of polymorphism for each gene across 14 *P. falciparum* isolates. (B) Amino acid sequences for each antigenic construct. Polymorphic residues are highlighted in red. (C) Amino acid sequence alignment of *mspdbl1* and *mspdbl2* allelic DBL region sequences showing regions of high conservation between the two genes. Symbol key: [*] denotes fully conserved residues, [:] denotes strongly similar conservative change (>0.5 in the Gonnet PAM 250 matrix), [.] denotes weakly similar conservative change (=<0.5 in the Gonnet PAM 250 matrix) (42-43).

Supplementary Figure S1A

MSPDBL1 (PF3D7_1035700)

```
                                   10        20        30        40        50        60
                          ....|....|....|....|....|....|....|....|....|....|....|....|
PF10_0348_3D7_ref seq     MKKIYSIFFSLFILNLHIYIKNIKCNDLINYNDSNLRNGLLNNSLDLTNGLNNKDNSFID
Pf10_0348_3D7             ............................................................
Pf10_0348_RO33            ............................................................
Pf10_0348_Fcr3            ............................................................
Pf10_0348_Wellcome        ............................................................
Pf10_0348_T996            ............................................................
Pf10_0348_T9102           ............................................................
Pf10_0348_K1              ............................................................
Pf10_0348_Dd2             ............................................................
Pf10_0348_D10             ............................................................
Pf10_0348_Fcc2            ............................................................
Pf10_0348_Palo_Alto       ............................................................
Pf10_0348_HB3             ............................................................
Pf10_0348_7G8             ............................................................
Pf10_0348_D6_             ............................................................

                                   70        80        90       100       110       120
                          ....|....|....|....|....|....|....|....|....|....|....|....|
PF10_0348_3D7_ref seq     SKIEEHENKSYQNKDNNISIVGQDVPITSVYSSKIINANDLEGNSIDDTKGLSVTNSGFD
Pf10_0348_3D7             ............................................................
Pf10_0348_RO33            ..............................D.............................
Pf10_0348_Fcr3            ..............................D.............................
Pf10_0348_Wellcome        ..............................D.............................
Pf10_0348_T996            ..............................D.............................
Pf10_0348_T9102           ..............................D.............................
Pf10_0348_K1              ..............................D.............................
Pf10_0348_Dd2             ..............................D.............................
Pf10_0348_D10             ..............................D.............................
Pf10_0348_Fcc2            ..............................D.............................
Pf10_0348_Palo_Alto       ..............................D.............................
Pf10_0348_HB3             ..............................D.............................
Pf10_0348_7G8             ..............................D.............................
Pf10_0348_D6_             ..............................D.............................

                                  130       140       150       160       170       180
                          ....|....|....|....|....|....|....|....|....|....|....|....|
PF10_0348_3D7_ref seq     DGSAFGGGLPFSGYSPLQGNHNKCPDENFCKGIKNVLSCPPKNSTGRNGDWISVAVKESS
Pf10_0348_3D7             ............................................................
Pf10_0348_RO33            ...................................A..................T.....
Pf10_0348_Fcr3            ................................................R...........
Pf10_0348_Wellcome        ................................................R...........
Pf10_0348_T996            ............................................................
Pf10_0348_T9102           ...................................A........................
Pf10_0348_K1              ...................................A........................
Pf10_0348_Dd2             ...................................A........................
Pf10_0348_D10             ...................................A........................
Pf10_0348_Fcc2            ...................................................A.SN.RNFL
Pf10_0348_Palo_Alto       ..........................K...N..Q..PN..L..F..TK...A.SN.RNFL
Pf10_0348_HB3             ..........................K...N..Q..PN..L.DF..TK...A.SN.RNFL
Pf10_0348_7G8             ..........................K...N..Q..PN..L.DF..TK...A.SN.RNFL
Pf10_0348_D6_             ......................................................N.....

                                  190       200       210       220       230       240
                          ....|....|....|....|....|....|....|....|....|....|....|....|
PF10_0348_3D7_ref seq     TTNKGVLVPPRRTKLCLR.NINKVWHRIKDEKNFKEEFVKVALGESNALMKHYKEKNLNA
Pf10_0348_3D7             ............................................................
Pf10_0348_RO33            ............................................................
Pf10_0348_Fcr3            ............................................................
Pf10_0348_Wellcome        ............................................................
Pf10_0348_T996            ............K.........Q.....................................
Pf10_0348_T9102           ............K.........Q.....................................
Pf10_0348_K1              ............................................................
Pf10_0348_Dd2             ............................................................
Pf10_0348_D10             ............................................................
Pf10_0348_Fcc2            .V..........KQM.F.I...NFPELK.T.GK.ENFIYSS.GS.AKQ.I.L.GNNTEK.
Pf10_0348_Palo_Alto       .V..........KQM.F.I...NFPELK.T.GK.ENFIYSS.GS.AKQ.I.L.GNNTEK.
Pf10_0348_HB3             .V..........KQM.F.I...NFPKLK.T.GK.ENFIYSS.GS.AKQ.I.L.GNNTEK.
Pf10_0348_7G8             .V..........KQM.F.I...NFPKLK.T.GK.ENFIYSS.GS.AKQ.I.L.GNNTEK.
Pf10_0348_D6_             ............KQM.F.I...NFPKLK.T.GK.ENFIYSS.GS.AKQ.I.L.GNNTEK.
```

```
                              250       260       270       280       290       300
                       ....|....|....|....|....|....|....|....|....|....|....|....|
PF10_0348_3D7_ref seq  LTAIKYGFSDMGDIIKGTDLIDYQITKNINRALDKILRNETSN.DKIKKRVDWWEANKSA
Pf10_0348_3D7          ............................................................
Pf10_0348_RO33         .....................................G......................
Pf10_0348_Fcr3         .....................................G......................
Pf10_0348_Wellcome     .....................................G......................
Pf10_0348_T996         .....................................G......................
Pf10_0348_T9102        .....................................G......................
Pf10_0348_K1           .....................................G......................
Pf10_0348_Dd2          .....................................G......................
Pf10_0348_D10          .....................................G......................
Pf10_0348_Fcc2         .Q.M....A.I.N.VQ.N.M..TPTSNKTKTY.EEV.GKQYK.VNDP.DAKT..IQ..HR
Pf10_0348_Palo_Alto    .Q.M....A.I.N.VQ.N.M..TPTSNKTKTY.EEV.GKQYK.VNDP.DAKT..IQ..HR
Pf10_0348_HB3          .Q.M....A.I.N.VQ.N.M..TPTSNKTKTY.EEV.GKQYK.VNDP.DAKT..IQ..HR
Pf10_0348_7G8          .Q.M....A.I.N.VQ.N.M..TPTSNKTKTY.EEV.GKQYK.VNDP.DAKT..IQ..HR
Pf10_0348_D6_          HQ..R.S.A.I.N..R.D.MM.TPTS.ETITY.E.V.KIYNE.N..P.DAKK..TE.RHH

                              310       320       330       340       350       360
                       ....|....|....|....|....|....|....|....|....|....|....|....|
PF10_0348_3D7_ref seq  FWDAFMCGYKVHIGNKPCPEHDNMDRIPQYLRWFREWGTYVCSEYKNKFEDVIKLCNIQQ
Pf10_0348_3D7          ............................................................
Pf10_0348_RO33         ..................................................N..E...VR.
Pf10_0348_Fcr3         ..................................................N..E...VR.
Pf10_0348_Wellcome     ..................................................N..E...VR.
Pf10_0348_T996         ............................................................
Pf10_0348_T9102        ............................................................
Pf10_0348_K1           ..................................................N..E...VR.
Pf10_0348_Dd2          ..................................................N..E...VR.
Pf10_0348_D10          ............................................................
Pf10_0348_Fcc2         V...M.............................................N..E...VR.
Pf10_0348_Palo_Alto    V...M.............................................N..E...VR.
Pf10_0348_HB3          V...M....QYEKKDNK.TGYG.IYD..................................
Pf10_0348_7G8          V...M....QYEKKDNK.TGYG.IYD..................................
Pf10_0348_D6_          V.E.M....QSAQKDNQ.TGYG.I.D..................................

                              370       380       390       400       410       420
                       ....|....|....|....|....|....|....|....|....|....|....|....|
PF10_0348_3D7_ref seq  FTNQDDSQLLEISKKDKCKEALKHYEEWVNRRRPEWKGQCDKFEKEKSKYEDTKSITAEK
Pf10_0348_3D7          ............................................................
Pf10_0348_RO33         I...N..............G...................................R...I
Pf10_0348_Fcr3         I...N..............G...................................R...I
Pf10_0348_Wellcome     I...N..............G...................................R...I
Pf10_0348_T996         ............................................................
Pf10_0348_T9102        ....N..............G...................................R...I
Pf10_0348_K1           I...N..............G...................................R...I
Pf10_0348_Dd2          I...N..............G...................................R...I
Pf10_0348_D10          ............................................................
Pf10_0348_Fcc2         I...N..............G...................................R...I
Pf10_0348_Palo_Alto    I...N..............G...................................R...I
Pf10_0348_HB3          ............................................................
Pf10_0348_7G8          ............................................................
Pf10_0348_D6_          ..............E...G...................................R...I

                              430       440       450       460       470       480
                       ....|....|....|....|....|....|....|....|....|....|....|....|
PF10_0348_3D7_ref seq  YLKEICSECDCKYKDLDNTFKEFKDNVTLLKAVIDNKKNQDSLTTTSLSTSINSVRDSSN
Pf10_0348_3D7          ............................................................
Pf10_0348_RO33         ............................................................
Pf10_0348_Fcr3         ............................................................
Pf10_0348_Wellcome     ............................................................
Pf10_0348_T996         ............................................................
Pf10_0348_T9102        .......................N....................................
Pf10_0348_K1           ............................................................
Pf10_0348_Dd2          ............................................................
Pf10_0348_D10          ................................................T.....G.....
Pf10_0348_Fcc2         .......................N....................................
Pf10_0348_Palo_Alto    .......................N....................................
Pf10_0348_HB3          ................................................T.....G.....
Pf10_0348_7G8          ................................................T.....G.....
Pf10_0348_D6_          ...QK.......................................................
```

```
                              490       500       510       520       530       540
                       ....|....|....|....|....|....|....|....|....|....|....|....|
PF10_0348_3D7_ref seq  LDQRGNITTSQGNSHRATVVQQVDQTNRLDNVNSVTQRGNNNYNNNLERGLGSGALPGTN
Pf10_0348_3D7          ............................................................
Pf10_0348_RO33         ......................A.....................................
Pf10_0348_Fcr3         ......................A.....................................
Pf10_0348_Wellcome     ......................A.....................................
Pf10_0348_T996         ......................A.....................................
Pf10_0348_T9102        ......................A.....................................
Pf10_0348_K1           ......................A.....................................
Pf10_0348_Dd2          ......................A.....................................
Pf10_0348_D10          .N....................A.....................................
Pf10_0348_Fcc2         ......................A.....................................
Pf10_0348_Palo_Alto    ......................A.....................................
Pf10_0348_HB3          .N....................A.....................................
Pf10_0348_7G8          .N....................A.....................................
Pf10_0348_D6_          ......................A.....................................

                              550       560       570       580       590       600
                       ....|....|....|....|....|....|....|....|....|....|....|....|
PF10_0348_3D7_ref seq  IITEEKYSLELIKLTSKDEEDIIKHNEDVREEIEEQQEDIEEDEEELENEGEE.......
Pf10_0348_3D7          ............................................................
Pf10_0348_RO33         ............................................................
Pf10_0348_Fcr3         ............................................................
Pf10_0348_Wellcome     ............................................................
Pf10_0348_T996         ............................................................
Pf10_0348_T9102        ............................................................
Pf10_0348_K1           ............................................................
Pf10_0348_Dd2          ............................................................
Pf10_0348_D10          ............................................................
Pf10_0348_Fcc2         ............................................................
Pf10_0348_Palo_Alto    ............................................................
Pf10_0348_HB3          ............................................................
Pf10_0348_7G8          ............................................................
Pf10_0348_D6_          ............................................................

                              610       620       630       640       650       660
                       ....|....|....|....|....|....|....|....|....|....|....|....|
PF10_0348_3D7_ref seq  ...........TKEEDDEEKNETNDTED...TDDTEDTEDIEEENKEKELSNQQQSEKKS
Pf10_0348_3D7          ............................................................
Pf10_0348_RO33         ............................................................
Pf10_0348_Fcr3         ............................................................
Pf10_0348_Wellcome     ............................................................
Pf10_0348_T996         ............................TED.............................
Pf10_0348_T9102        ............................................................
Pf10_0348_K1           .............................................E..............
Pf10_0348_Dd2          .............................................E..............
Pf10_0348_D10          .............................................E..............
Pf10_0348_Fcc2         ............................................................
Pf10_0348_Palo_Alto    ............................................................
Pf10_0348_HB3          ............................................................
Pf10_0348_7G8          ............................................................
Pf10_0348_D6_          ............................TED.............................

                              670       680       690       700       710       720
                       ....|....|....|....|....|....|....|....|....|....|....|....|
PF10_0348_3D7_ref seq  ISKVDEDSYRILSVSYKDNNEVKNVAESIVKKLFSLFNDNNNLETIFKGLTEDMTDLFQK
Pf10_0348_3D7          ............................................................
Pf10_0348_RO33         ............................................................
Pf10_0348_Fcr3         ............................................................
Pf10_0348_Wellcome     ............................................................
Pf10_0348_T996         ............................................................
Pf10_0348_T9102        ............................................................
Pf10_0348_K1           ............................................................
Pf10_0348_Dd2          ............................................................
Pf10_0348_D10          ............................................................
Pf10_0348_Fcc2         ............................................................
Pf10_0348_Palo_Alto    ............................................................
Pf10_0348_HB3          ............................................................
Pf10_0348_7G8          ............................................................
Pf10_0348_D6_          ............................................................
```

MSPDBL2 (PF3D7_1036300)

```
                                  10        20        30        40        50        60
                          ....|....|....|....|....|....|....|....|....|....|....|....|
PF10_0355_3D7_ref seq     MIYILSIVFYIFFLHIDIYVNIYSTCFVVNEGNPNLRNNIINDDELKGKAYNNTIDANNQ
Pf10_0355_3D7             ............................................................
Pf10_0355_RO33            ............................................................
Pf10_0355_Fcr3            ............................................................
Pf10_0355_Wellcome        ............................................................
Pf10_0355_T996            ............................................................
Pf10_0355_T9102           ............................................................
Pf10_0355_K1              ............................................................
Pf10_0355_Dd2             ............................................................
Pf10_0355_D10             ............................................................
Pf10_0355_Fcc2            ............................................................
Pf10_0355_Palo_Alto       ............................................................
Pf10_0355_HB3             ............................................................
Pf10_0355_7G8             ............................................................
Pf10_0355_D6              ............................................................

                                  70        80        90       100       110       120
                          ....|....|....|....|....|....|....|....|....|....|....|....|
PF10_0355_3D7_ref seq     NIEYNKNLKHNVNSSHISKFSDIMDQEDKGDNENSHDIKFEE.KKNINKSLDAESNYGIN
Pf10_0355_3D7             ............................................................
Pf10_0355_RO33            ............................................................
Pf10_0355_Fcr3            ..........................................V.................
Pf10_0355_Wellcome        ..........................................V.................
Pf10_0355_T996            ..........................................V.................
Pf10_0355_T9102           ............................................................
Pf10_0355_K1              ..........................................V.................
Pf10_0355_Dd2             ............................................................
Pf10_0355_D10             ............................................................
Pf10_0355_Fcc2            ...................................Y........................
Pf10_0355_Palo_Alto       ............................................................
Pf10_0355_HB3             ............................................................
Pf10_0355_7G8             ............................................................
Pf10_0355_D6              ...................................Y........................

                                 130       140       150       160       170       180
                          ....|....|....|....|....|....|....|....|....|....|....|....|
PF10_0355_3D7_ref seq     EISITGNDSNSDNSNQNIF.PDGSELAGGIPRSIYTINLG.FNKCPTEEICKDFSNLPQC
Pf10_0355_3D7             ............................................................
Pf10_0355_RO33            ..............................AP...SN....P....V.K.....GP....
Pf10_0355_Fcr3            ........IT.GAR.NG.IS..D.AFG..L.F.D.SHLE.NHY...DKNF.NGIQ.V.N.
Pf10_0355_Wellcome        ........IT.GAR.NG.IS..D.AFG..L.F.D.SHLE.NHY...DKNF.NGIQ.V.N.
Pf10_0355_T996            ........IT.GAR.NG.IS..D.AFG..L.F.D.SHLE.NHY...DKNF.NGIQ.V.N.
Pf10_0355_T9102           ............................................................
Pf10_0355_K1              ........IT.GAR.NG.IS..D.AFG..L.F.D.SHLE.NHY...DKNF.NGIQ.V.N.
Pf10_0355_Dd2             ..............................AP...SN....P....V.K.....GP....
Pf10_0355_D10             ............................................................
Pf10_0355_Fcc2            ............................................................
Pf10_0355_Palo_Alto       ............................................................
Pf10_0355_HB3             ..............................AP...SN....P....V.K.....GP....
Pf10_0355_7G8             ............................................................
Pf10_0355_D6              ............................................................

                                 190       200       210       220       230       240
                          ....|....|....|....|....|....|....|....|....|....|....|....|
PF10_0355_3D7_ref seq     RKNVHE..RNNWLGSSVKNFSSDNKGVLVPPRRQSLCLRITLQDFRTKKKKEGDFEKFIY
Pf10_0355_3D7             ............................................................
Pf10_0355_RO33            ..D..........S.N..K....Y..........F........N..A...NK........
Pf10_0355_Fcr3            PLKDFTGTKGD.AS.N.R..LTV..........KQM.F..NINN.PKL..T..K..N...
Pf10_0355_Wellcome        PLKDFTGTKGD.AS.N.R..LTV..........KQM.F..NINN.PKL..T..K..N...
Pf10_0355_T996            PLKDFTGTKGD.AS.N.R..LTV..........KQM.F..NINN.PKL..T..K..N...
Pf10_0355_T9102           ............................................................
Pf10_0355_K1              PLKDFTGTKGD.AS.N.R..LTV..........KQM.F..NINN.PKL..T..K..N...
Pf10_0355_Dd2             ..D..........S.N..K.........................................
Pf10_0355_D10             ............................................................
Pf10_0355_Fcc2            ...........................................N.HA...NK........
Pf10_0355_Palo_Alto       ............................................................
Pf10_0355_HB3             ..D..........S.N..K.........................................
Pf10_0355_7G8             ............................................................
Pf10_0355_D6              ...........................................N.HA...NK........
```

```
                                 250       260       270       280       290       300
                          ....|....|....|....|....|....|....|....|....|....|....|....|
PF10_0355_3D7_ref seq     SYASSEARKLRTIHNNNLEKAHQAIRYSFADIGNIIRGDDMMDTPTSKETITYLEKVLKI
Pf10_0355_3D7             ............................................................
Pf10_0355_RO33            .......K........IS...L...K..........K...LL.....NN.K....E...L
Pf10_0355_Fcr3            .S.G...KQ.IKLYG..T...L..MK.G.......VQ.N..I.....NK.K....E..GK
Pf10_0355_Wellcome        .S.G...KQ.IKLYG..T...L..MK.G.......VQ.N..I.....NK.K....E..GK
Pf10_0355_T996            .S.G...KQ.IKLYG..T...L..MK.G.......VQ.N..I.....NK.K....E..GK
Pf10_0355_T9102           ............................................................
Pf10_0355_K1              .S.G...KQ.IKLYG..T...L..MK.G.......VQ.N..I.....NK.K....E..GK
Pf10_0355_Dd2             ............................................................
Pf10_0355_D10             .......K........IS...L...K..........K...LL.....NN.K....E...L
Pf10_0355_Fcc2            .......K........IS...L...K..........K...LL.....NN.K....E...L
Pf10_0355_Palo_Alto       ............................................................
Pf10_0355_HB3             .......K........IS...L...K..........K...LL.....NN.K....E...L
Pf10_0355_7G8             ............................................................
Pf10_0355_D6              .......K........IS...L...K..........K...LL.....NN.K....E...L

                                 310       320       330       340       350       360
                          ....|....|....|....|....|....|....|....|....|....|....|....|
PF10_0355_3D7_ref seq     YNENNDKPKDAKKWWTENRHHVWEAMMCGYQSAQKDNQCTGYGNIDDIPQFLRWFREWGT
Pf10_0355_3D7             ............................................................
Pf10_0355_RO33            H.K..E.T...........Y...........NEK...E..V...................
Pf10_0355_Fcr3            HYK.VND.....T..IQ.K.R..D.......YEK...K.......Y.............I
Pf10_0355_Wellcome        HYK.VND.....T..IQ.K.R..D.......YEK...K.......Y.............I
Pf10_0355_T996            QYK.VND.....T..IQ.K.R..D.......YEK...K.......Y..............
Pf10_0355_T9102           ............................................................
Pf10_0355_K1              QYK.VND.....T..IQ.K.R..D.......YEK...K.......Y.............I
Pf10_0355_Dd2             ............................................................
Pf10_0355_D10             H.K..E.T...........Y...........NEK...E..V...................
Pf10_0355_Fcc2            H.K..E.T...........Y...........NEK...E..V...................
Pf10_0355_Palo_Alto       ............................................................
Pf10_0355_HB3             H.K..E.T...........Y...........NEK...E..V...................
Pf10_0355_7G8             ............................................................
Pf10_0355_D6              H.K..E.T...........Y...........NEK...E..V...................

                                 370       380       390       400       410       420
                          ....|....|....|....|....|....|....|....|....|....|....|....|
PF10_0355_3D7_ref seq     YVCEESEKNMNTLKAVCFPKQPRTEANPALTVHENEMCSSTLKKYEEWYNKRKTEWTEQS
Pf10_0355_3D7             ............................................................
Pf10_0355_RO33            .......N..K..........L...T.S.M..TQKGT......N.........N......
Pf10_0355_Fcr3            ............................................................
Pf10_0355_Wellcome        ............................................................
Pf10_0355_T996            ............................................................
Pf10_0355_T9102           .......N..K..........L...T.S.M..TQKGT......N.........N......
Pf10_0355_K1              ............................................................
Pf10_0355_Dd2             ............................................................
Pf10_0355_D10             .......N..K..........L...T.S.M..TQKGT......N.........N......
Pf10_0355_Fcc2            .......N..K..........L...T.S.M..TQKGT......N.........N......
Pf10_0355_Palo_Alto       ............................................................
Pf10_0355_HB3             .......N..K..........L...T.S.M..TQKGT......N.........N......
Pf10_0355_7G8             .......N..K..........L...T.S.M..TQKGT......N.........N......
Pf10_0355_D6              .......N..K..........L...T.S.M..TQKGT......N.........N......

                                 430       440       450       460       470       480
                          ....|....|....|....|....|....|....|....|....|....|....|....|
PF10_0355_3D7_ref seq     IKYNNDKINYTDIKTLSPSEYLIEKCPECKCTKKNLQDVFELTFDGKALLEKLKKEESPV
Pf10_0355_3D7             ............................................................
Pf10_0355_RO33            ....K..TK.K..EN....N...K........E...H....H........K..EE....A
Pf10_0355_Fcr3            ............................................................
Pf10_0355_Wellcome        ............................................................
Pf10_0355_T996            ............................................................
Pf10_0355_T9102           ....K..TK.K..EN....N...K........E...H....H........K..EE....A
Pf10_0355_K1              ............................................................
Pf10_0355_Dd2             ............................................................
Pf10_0355_D10             ....K..TK.K..EN....N...K........E...H....H........K..EE....A
Pf10_0355_Fcc2            ....K..TK.K..EN....N...K........E...H....H........K..EE....A
Pf10_0355_Palo_Alto       ............................................................
Pf10_0355_HB3             ....K..TK.K..EN....N...K........E...H....H........K..EE....A
Pf10_0355_7G8             ....K..TK.K..EN....N...K........E...H....H........K..EE....A
Pf10_0355_D6              ....K..TK.K..EN....N...K........E...H....H........K..EE....A
```

```
                           490       500       510       520       530       540
                       ....|....|....|....|....|....|....|....|....|....|....|....|
PF10_0355_3D7_ref seq  SNSVNALPEPGQITLPDPSLKQTTQQENQPVVETPVTTAVINEHQGQTEPNKGDNNNERE
Pf10_0355_3D7          ............................................................
Pf10_0355_RO33         .D..RTST.S.P..V..S..N.........G..K.L..V.M.......D..TD..SG.T.
Pf10_0355_Fcr3         ............................................................
Pf10_0355_Wellcome     ............................................................
Pf10_0355_T996         ............................................................
Pf10_0355_T9102        .D..RTST.S.P..V..S..N.........G..K.L..V.M.......D..TD..SG.T.
Pf10_0355_K1           ............................................................
Pf10_0355_Dd2          ............................................................
Pf10_0355_D10          .D..RTST.S.P..V..S..N.........G..K.L..V.M.......D..TD..SG.T.
Pf10_0355_Fcc2         .D..RTST.S.P..V..S..N.........G..K.L..V.M.......D..TD..SG.T.
Pf10_0355_Palo_Alto    ............................................................
Pf10_0355_HB3          .D..RTST.S.P..V..S..N.........G..K.L..V.M.......D..TD..SG.T.
Pf10_0355_7G8          .D..RTST.S.P..V..S..N.........G..K.L..V.M.......D..TD..SG.T.
Pf10_0355_D6           .D..RTST.S.P..V..S..N.........G..K.L..V.M.......D..TD..SG.T.

                           550       560       570       580       590       600
                       ....|....|....|....|....|....|....|....|....|....|....|....|
PF10_0355_3D7_ref seq  NHESNVGSIQEVNQGSVSEESHSKTIDPSKIDDRLELSSGSSSLEQHSKEDVKKGCALEL
Pf10_0355_3D7          ............................................................
Pf10_0355_RO33         HNK....................................................S....
Pf10_0355_Fcr3         ............................................................
Pf10_0355_Wellcome     ............................................................
Pf10_0355_T996         ............................................................
Pf10_0355_T9102        HNK....................................................S....
Pf10_0355_K1           .......................................................S....
Pf10_0355_Dd2          .......................................................S....
Pf10_0355_D10          HNK....................................................S....
Pf10_0355_Fcc2         HNK....................................................S....
Pf10_0355_Palo_Alto    ............................................................
Pf10_0355_HB3          HNK....................................................ST...
Pf10_0355_7G8          HNK....................................................S....
Pf10_0355_D6           HNK....................................................S....

                           610       620       630       640       650       660
                       ....|....|....|....|....|....|....|....|....|....|....|....|
PF10_0355_3D7_ref seq  VPLSLSDIEQIANESEDVLEEIEEEINTDGEIEYITEEEIKEDIEEETEEDIEEET....
Pf10_0355_3D7          ............................................................
Pf10_0355_RO33         ........................................................EEET
Pf10_0355_Fcr3         ............................................................
Pf10_0355_Wellcome     ............................................................
Pf10_0355_T996         ............................................................
Pf10_0355_T9102        ............................................................
Pf10_0355_K1           ............................................................
Pf10_0355_Dd2          ............................................................
Pf10_0355_D10          ........................................................EEET
Pf10_0355_Fcc2         ....................................D...................EEET
Pf10_0355_Palo_Alto    ............................................................
Pf10_0355_HB3          ........................................................EEET
Pf10_0355_7G8          ..............................................IK........EEDI
Pf10_0355_D6           ........................................................EEET

                           670       680       690       700       710       720
                       ....|....|....|....|....|....|....|....|....|....|....|....|
PF10_0355_3D7_ref seq  EEETEEETEEEADEETVKEIEDKPEQEIKNKSLEEKQIDKNTDTSEKKGFNNSEKDEKAR
Pf10_0355_3D7          ............................................................
Pf10_0355_RO33         ..................V.........................................
Pf10_0355_Fcr3         ............................................................
Pf10_0355_Wellcome     ............................................................
Pf10_0355_T996         ............................................................
Pf10_0355_T9102        ..................V.........................................
Pf10_0355_K1           ............................................................
Pf10_0355_Dd2          ............................................................
Pf10_0355_D10          ............................................................
Pf10_0355_Fcc2         ............................................................
Pf10_0355_Palo_Alto    ............................................................
Pf10_0355_HB3          ............................................................
Pf10_0355_7G8          ............................................................
Pf10_0355_D6           ............................................................
```

```
                                 730       740       750       760       770
                         ....|....|....|....|....|....|....|....|....|....|..
PF10_0355_3D7_ref seq    NLISKNYKNYNELDKNVHTLVNSIISLLEEGNGSDSTLNSLSKDITNLFKN.
Pf10_0355_3D7            ....................................................
Pf10_0355_RO33           ....................................................
Pf10_0355_Fcr3           ....................................................
Pf10_0355_Wellcome       ....................................................
Pf10_0355_T996           ....................................................
Pf10_0355_T9102          ....................................................
Pf10_0355_K1             ....................................................
Pf10_0355_Dd2            ....................................................
Pf10_0355_D10            ....................................................
Pf10_0355_Fcc2           ....................................................
Pf10_0355_Palo_Alto      ....................................................
Pf10_0355_HB3            ....................................................
Pf10_0355_7G8            ....................................................
Pf10_0355_D6             ....................................................
```

SURFIN$_{4.2}$ (PF3D7_0424400)

```
                                      10        20        30        40        50        60
                              ....|....|....|....|....|....|....|....|....|....|....|....|
PFD1160w_3D7_ref_seq_exon_1   MLFVVELDSRLEKSADKRISVERFRKIFEIYVEDKLEELKRSGSEKYDKDCRDFNYFIDD
PFD1160w_3D7                  ............................................................
PFD1160w_RO33                 .........................E..............K................M..
PFD1160w_Fcr3                 .........................E...............................M..
PFD1160w_Wellcome             .........................E...............................M..
PFD1160w_T996                 .........................E...............................M..
PFD1160w_T9102                .........................E...............................M..
PFD1160w_K1                   .........................E...............................M..
PFD1160w_Dd2                  .........................E...............................M..
PFD1160w_D10                  .........................E...............................M..
PFD1160w_FCC2                 .........................E...............................M..
PFD1160w_Palo_Alto            .........................................................M..
PFD1160w_HB3                  .........................................................M..
PFD1160w_7G8                  .........................E...............................M..
PFD1160w_D6                   .........................................................M..

                                      70        80        90       100       110       120
                              ....|....|....|....|....|....|....|....|....|....|....|....|
PFD1160w_3D7_ref_seq_exon_1   VKDVFINNDLVKIPVKVRKSIWETHVDKNLPKLMKNTTSCKCIRKEHNYNKEYRDMTRTL
PFD1160w_3D7                  ............................................................
PFD1160w_RO33                 ...............E...............T.........................Q..
PFD1160w_Fcr3                 ...............E............................................
PFD1160w_Wellcome             ...............E............................................
PFD1160w_T996                 ...............E...............T.........................Q..
PFD1160w_T9102                ...............E...............T.................K..........
PFD1160w_K1                   ...............E...............T............................
PFD1160w_Dd2                  ...............E...............T.........................Q..
PFD1160w_D10                  ...............E...............T.........................Q..
PFD1160w_FCC2                 ...............E............................................
PFD1160w_Palo_Alto            ..............FE...............T.........................Q..
PFD1160w_HB3                  ...............E...............T.................K..........
PFD1160w_7G8                  ........N......D...............T...K........................
PFD1160w_D6                   ...............................T............................

                                     130       140       150       160       170       180
                              ....|....|....|....|....|....|....|....|....|....|....|....|
PFD1160w_3D7_ref_seq_exon_1   EDFCEEKTRKLEIIYQKDYDESLYVNFNEWINKKKEDILKEYEKLSNKDKYKHLLKISET
PFD1160w_3D7                  ............................................................
PFD1160w_RO33                 .........MR...D.........M...K...............................
PFD1160w_Fcr3                 .........M....D.............K...................NE......V...
PFD1160w_Wellcome             .........M....D.............K...................NE......V...
PFD1160w_T996                 .........MR...D.............K...................NE..........
PFD1160w_T9102                ..............D.............K....................E..........
PFD1160w_K1                   .........M....D..........D..K...................NE..........
PFD1160w_Dd2                  .........MR...D.........M...K...............................
PFD1160w_D10                  .........MR...D.........M...K...............................
PFD1160w_FCC2                 .........M....D.N...........K.............................K.
PFD1160w_Palo_Alto            .......S.M....D.E.......M........................E..........
PFD1160w_HB3                  ..............D.E.......M........................E..........
PFD1160w_7G8                  .........M....D.M...........K...............................
PFD1160w_D6                   ..............D.............K.............K......E......V...

                                     190       200       210       220       230       240
                              ....|....|....|....|....|....|....|....|....|....|....|....|
PFD1160w_3D7_ref_seq_exon_1   CDLNHVDKLFLNISSEDMKKMKEDVKKQHLEVKVRPPVNEPIDDRGKEDALSRGGKSIIL
PFD1160w_3D7                  ............................................................
PFD1160w_RO33                 ................N........T.K.R......L.......................
PFD1160w_Fcr3                 ....................N....T.K.R......L.......................
PFD1160w_Wellcome             ....................N....T.K.R......L.......................
PFD1160w_T996                 ............................................................
PFD1160w_T9102                ....................N....T.K.R......L................R......
PFD1160w_K1                   ....................N....T.K.R......L.......................
PFD1160w_Dd2                  ....................N....T.K.R......L.......................
PFD1160w_D10                  ....................N....T.K.R......L.......................
PFD1160w_FCC2                 ....................N....T.K.R...................D.I....R...
PFD1160w_Palo_Alto            ....N.................................K.....................
PFD1160w_HB3                  .....................................................E......
PFD1160w_7G8                  ................N................................D..........
PFD1160w_D6                   ..........P.....................N...........................
```

```
                                      250       260       270       280       290       300
                             ....|....|....|....|....|....|....|....|....|....|....|....|
PFD1160w_3D7_ref_seq_exon_1  NKEENSPTEEITTEYNPVSEMGVGTTIAHSEPGPKTVNTEVRNVLRSDGKISDQGSQKSP
PFD1160w_3D7                 ............................................................
PFD1160w_RO33                ............................................................
PFD1160w_Fcr3                ............................................................
PFD1160w_Wellcome            ............................................................
PFD1160w_T996                ...............................S............................
PFD1160w_T9102               ........A......................................G........EI..
PFD1160w_K1                  ...............................S...............G............
PFD1160w_Dd2                 ...............................S...............G............
PFD1160w_D10                 ...............................S...............G............
PFD1160w_FCC2                ............................................................
PFD1160w_Palo_Alto           ............................................................
PFD1160w_HB3                 ............................................................
PFD1160w_7G8                 ............................................................
PFD1160w_D6                  ............................................................

                                      310       320       330       340       350       360
                             ....|....|....|....|....|....|....|....|....|....|....|....|
PFD1160w_3D7_ref_seq_exon_1  PKELSNKQMTPAQRKNVPHFVERRGYGNSHVRGNALKKISNGDDNYKSPSSNYIEVDCAE
PFD1160w_3D7                 ............................................................
PFD1160w_RO33                ............................................................
PFD1160w_Fcr3                ..........................................................D.
PFD1160w_Wellcome            ..........................................................D.
PFD1160w_T996                ............................................................
PFD1160w_T9102               ..A.......................................................D.
PFD1160w_K1                  ..................................S.........................
PFD1160w_Dd2                 ............................................................
PFD1160w_D10                 ............................................................
PFD1160w_FCC2                ............................................................
PFD1160w_Palo_Alto           ..................................S.........................
PFD1160w_HB3                 ............................................................
PFD1160w_7G8                 ..........................................................D.
PFD1160w_D6                  ............................................................

                                      370       380       390       400       410       420
                             ....|....|....|....|....|....|....|....|....|....|....|....|
PFD1160w_3D7_ref_seq_exon_1  DKYFLLEDGTNQSENSCKTKYNYFVSNDYDGTGSAIYSTDQVPSREEIKSPDSLSTLDAR
PFD1160w_3D7                 ............................................................
PFD1160w_RO33                ...........P.........V......................................
PFD1160w_Fcr3                ..FL.................D.....................RM......A........
PFD1160w_Wellcome            ..FL.................D.....................RM......A........
PFD1160w_T996                ...........P...........S....................................
PFD1160w_T9102               ..FL........................................................
PFD1160w_K1                  ..FL...............I.D.....................RM......A........
PFD1160w_Dd2                 ...........P.........D...................................NS.
PFD1160w_D10                 ...........P.........D...................................NS.
PFD1160w_FCC2                ...........P.........V......................................
PFD1160w_Palo_Alto           ...................I.D.....................RM......A........
PFD1160w_HB3                 ...........P.........D...................................NS.
PFD1160w_7G8                 ...........P...........S....................................
PFD1160w_D6                  ...........P.........D...................................NS.

                                      430       440       450       460       470       480
                             ....|....|....|....|....|....|....|....|....|....|....|....|
PFD1160w_3D7_ref_seq_exon_1  GSTHNLNVSNEGNPLEGGEEKNNVKISEQNGRNLESSVGTDKGSDKNEEEVAATCDPNDR
PFD1160w_3D7                 ............................................................
PFD1160w_RO33                ..................G........................L.........I.....G
PFD1160w_Fcr3                .............T..............................VEK........G...G
PFD1160w_Wellcome            .............T..............................VEK........G...G
PFD1160w_T996                ..........KR.......G....T.N...V......................I.....G
PFD1160w_T9102               .........................................................D..
PFD1160w_K1                  ........................T.N............................G...G
PFD1160w_Dd2                 V.........KR.......G........................VEK........G...G
PFD1160w_D10                 V.........KR.......G........................VEK........G...G
PFD1160w_FCC2                ...........................................................G
PFD1160w_Palo_Alto           ........................T.N.................................
PFD1160w_HB3                 V..........................................................G
PFD1160w_7G8                 ..........KR.......G....T.N...V..........................D..
PFD1160w_D6                  V..................G....T.N...V......................I.....A
```

```
                                         490       500       510       520       530       540
                               ....|....|....|....|....|....|....|....|....|....|....|....|
PFD1160w_3D7_ref_seq_exon_1    NCFDGRYINVDYIRGLLKGKRSGSDGRSNIKHIISNNFDDSNMIFPTFNFDFDILKAEEE
PFD1160w_3D7                   ............................................................
PFD1160w_RO33                  ..I.ANQ.P.E.L.N.SR.E...L..S..MA.F..T..GGN.IFSS.G.L....VRPAR.
PFD1160w_Fcr3                  D.I.ANQ.P.E.L.K.SR.E...L..S..MA.F..TH.GGN.IFSS.G.L....VRPAR.
PFD1160w_Wellcome              D.I.ANQ.P.E.L.K.SR.E...L..S..MA.F..TH.GGN.IFSS.G.L....VRPAR.
PFD1160w_T996                  A.I.ANQ.P.E.L.N.............................................
PFD1160w_T9102                 ..V.VTK.S.E.L.N.SR.E...L..S..MAQF..TH.G.H.IFSS.D.LY...VRPAK.
PFD1160w_K1                    D.I.ANQ.P.E.L.N..R.E...L..S..MA.F..T..GGN.IFSS.D.LY...VRPAR.
PFD1160w_Dd2                   D.I.ANQ.P.E.L.K.SR.E...L..S..MA.F..TH.GGN.IFSS.D.L....VRPAKG
PFD1160w_D10                   D.I.ANQ.P.E.L.K.SR.E...L..S..MA.F..TH.GGN.IFSS.D.L....VRPAKG
PFD1160w_FCC2                  D........F..................................................
PFD1160w_Palo_Alto             ............................................................
PFD1160w_HB3                   D........F..................................................
PFD1160w_7G8                   ..V.ANQ.A.E.L.N.SR.E...L..S..MAQF..TH.G.H.IFSS.D.LY.........
PFD1160w_D6                    D...ANQ.LL..FK..SE..G..L..S..MERLTLKY.R.N.IFSS.G.L....VRPAK.

                                         550       560       570       580       590       600
                               ....|....|....|....|....|....|....|....|....|....|....|....|
PFD1160w_3D7_ref_seq_exon_1    VLPVNNSDIIYGHEEVEETTQEGASIFEKHSHTSSQQNDSSASGNKYRMLSTTMELPNQQ
PFD1160w_3D7                   ............................................................
PFD1160w_RO33                  ...ESK.K..HE.Q.M....E.....I.R..R....N..AGT.DYM..I......F..EK
PFD1160w_Fcr3                  ...ESK.K..HE.Q.M....E.....I.R..R....N..ADT.DYM..I...........
PFD1160w_Wellcome              ...ESK.K..HE.Q.M....E.....I.R..R....N..ADT.DYM..I...........
PFD1160w_T996                  ............................................................
PFD1160w_T9102                 ...ESK.K..HE.Q.M....E.....I.R.......N..AGT.DYM..I...........
PFD1160w_K1                    ...ESK.K..HE.Q.M....E.....I.R..R....N..AGT.DYM..I......F..EK
PFD1160w_Dd2                   ...ESK.N..HE.Q.M............................................
PFD1160w_D10                   ...ESK.N..HE.Q.M............................................
PFD1160w_FCC2                  ..........................I.R.......................S.......
PFD1160w_Palo_Alto             ....H........Q.M............................................
PFD1160w_HB3                   ..........................I.R...............................
PFD1160w_7G8                   ............................................................
PFD1160w_D6                    ...ESK.K..HE.Q............I.R..R....N..AGT.DYI.......I.F..EK

                                         610       620       630       640       650       660
                               ....|....|....|....|....|....|....|....|....|....|....|....|
PFD1160w_3D7_ref_seq_exon_1    EVFGLYSPVSRTLDSAMSFLRSIISLSSAPVSRSEGQSKESKRVEISTTVQDPIGYRTSP
PFD1160w_3D7                   ............................................................
PFD1160w_RO33                  .T........Q.F.R.LN....VFIS..S...Q.K...............KG........
PFD1160w_Fcr3                  ....................KN.FNS..S...Q.K......QLAQ......G........
PFD1160w_Wellcome              ....................KN.FNS..S...Q.K......QLAQ......G........
PFD1160w_T996                  ............FGR.LN.......F..................................
PFD1160w_T9102                 ....................KR.FIS..S...Q.K......QLAQ......G........
PFD1160w_K1                    .T........Q.F.R.LN....VFIS..S...Q.K......QLAQ.....RG....KN.S
PFD1160w_Dd2                   ....................KN.FNS..S...QRQ......QLAQ......G........
PFD1160w_D10                   ....................KN.FNS..S...QRQ......QLAQ......G........
PFD1160w_FCC2                  ..........Q.FGR.LN.......F..................................
PFD1160w_Palo_Alto             .........................F..................................
PFD1160w_HB3                   ............FG...N.......F..................................
PFD1160w_7G8                   .................N.......F........K......QLAQ...............
PFD1160w_D6                    .T........Q.FG.V....KN.FIS..S...Q.K......QLAQ.P.M.KG....KN.S

                                         670       680       690       700       710       720
                               ....|....|....|....|....|....|....|....|....|....|....|....|
PFD1160w_3D7_ref_seq_exon_1    LQMNAHSVGAGINVSSILSMLGLSSGQVRRSGGQGSETYIVGTSQSGFHKNEVIPSIKDK
PFD1160w_3D7                   ............................................................
PFD1160w_RO33                  ............................................................
PFD1160w_Fcr3                  ............................................................
PFD1160w_Wellcome              ............................................................
PFD1160w_T996                  ............................................................
PFD1160w_T9102                 ............................................................
PFD1160w_K1                    ............................................................
PFD1160w_Dd2                   ............................................................
PFD1160w_D10                   ............................................................
PFD1160w_FCC2                  ............................................................
PFD1160w_Palo_Alto             ............................................................
PFD1160w_HB3                   ............................................................
PFD1160w_7G8                   ............................................................
PFD1160w_D6                    ............................................................
```

```
                                       730       740       750       760
                             ....|....|....|....|....|....|....|....|....
PFD1160w_3D7_ref_seq_exon_1  SGKTQIVSNEKGGIFSKGITSMMSSLPVALVTFVFLFMFLVFNK
PFD1160w_3D7                 ............................................
PFD1160w_RO33                ............................................
PFD1160w_Fcr3                ............................................
PFD1160w_Wellcome            ............................................
PFD1160w_T996                ............................................
PFD1160w_T9102               ............................................
PFD1160w_K1                  ............................................
PFD1160w_Dd2                 ............................................
PFD1160w_D10                 ............................................
PFD1160w_FCC2                ............................................
PFD1160w_Palo_Alto           ............................................
PFD1160w_HB3                 ............................................
PFD1160w_7G8                 ............................................
PFD1160w_D6                  ............................................
```

Supplementary Figure S1B

MSPDBL1 (Gene id: PF3D7_1035700; also referred to as MSP3.4)

Allelic antigens

3D7

NHNKCPDENFCKGIKNVLSCPPKNSTGRNGDWISVAVKESSTTNKGVLVPPRRTKLCLRNINKVWHRIKDEKNFKEEFVKVALGESNALMKHYKEKNLNALTAIKYGFSDMGDIIKGT
DLIDYQITKNINRALDKILRNETSNDKIKKRVDWWEANKSAFWDAFMCGYKVHIGNKPCPEHDNMDRIPQYLRWFREWGTYVCSEYKNKFEDVIKLCNIQQFTNQDDSQLLEISKKD
KCKEALKHYEEWVNRRRPEWKGQCDKFEKEKSKYEDTKSITAEKYLKEICSECDCKYKDLDNTFKEF

7G8

NHNKCPDKNFCNGIQNVPNCPLKDFTGTKGDWASSNVRNFLTVNKGVLVPPRRKQMCFRININNFPKLKKTEGKFENFIYSSAGSEAKQLIKLYGNNTEKALQAMKYGFADIGNIVQ
GNDMIDTPTSNKTKTYLEEVLGKQYKNVNDPKDAKTWWIQNKHRVWDAMMCGYQYEKKDNKCTGYGNIYDIPQYLRWFREWGTYVCSEYKNKFEDVIKLCNIQQFTNQDDSQL
LEISKKDKCKEALKHYEEWVNRRRPEWKGQCDKFEKEKSKYEDTKSITAEKYLKEICSECDCKYKDLDNTFKEF

Fcc2

NHNKCPDENFCKGIKNVLSCPPKNSTGRNGDWASSNVRNFLTVNKGVLVPPRRKQMCFRININNFPELKKTEGKFENFIYSSAGSEAKQLIKLYGNNTEKALQAMKYGFADIGNIVQ
GNDMIDTPTSNKTKTYLEEVLGKQYKNVNDPKDAKTWWIQNKHRVWDAMMCGYKVHIGNKPCPEHDNMDRIPQYLRWFREWGTYVCSEYKNKFENVIELCNVRQITNQNDSQ
LLEISKKDKCKGALKHYEEWVNRRRPEWKGQCDKFEKEKSKYEDTKSRTAEIYLKEICSECDCKYKDLDNTFKEF

Palo Alto

NHNKCPDKNFCNGIQNVPNCPLKNFTGTKGDWASSNVRNFLTVNKGVLVPPRRKQMCFRININNFPELKKTEGKFENFIYSSAGSEAKQLIKLYGNNTEKALQAMKYGFADIGNIVQ
GNDMIDTPTSNKTKTYLEEVLGKQYKNVNDPKDAKTWWIQNKHRVWDAMMCGYKVHIGNKPCPEHDNMDRIPQYLRWFREWGTYVCSEYKNKFENVIELCNVRQITNQNDSQ
LLEISKKDKCKGALKHYEEWVNRRRPEWKGQCDKFEKEKSKYEDTKSRTAEIYLKEICSECDCKYKDLDNTFKEF

D6

NHNKCPDENFCKGIKNVLSCPPKNSTGRNGDWISVNVKESSTTNKGVLVPPRRKQMCFRININNFPKLKKTEGKFENFIYSSAGSEAKQLIKLYGNNTEKAHQAIRYSFADIGNIIRGDD MMDTPTSKETITYLEKVLKIYNENNDKPKDAKKWWTENRHHVWEAMMCGYQSAQKDNQCTGYGNIDDIPQYLRWFREWGTYVCSEYKNKFEDVIKLCNIQQFTNQDDSQLLEIS KEDKCKGALKHYEEWVNRRRPEWKGQCDKFEKEKSKYEDTKSRTAEIYLKQKCSECDCKYKDLDNTFKEF

MSPDBL1 (Gene id: PF3D7_1035700; also referred to as MSP3.4)

Conserved antigens (minimal polymorphism)

N-terminal

DSNLRNGLLNNSLDLTNGLNNKDNSFIDSKIEEHENKSYQNKDNNISIVGQDVPITSVYSSKIINANDLEGNSIDDTKGLSVTNSGFDDGSAFGGGLPFSGYSPLQ

C-terminal

QRGNITTSQGNSHRATVVQQVDQTNRLDNVNSVTQRGNNNYNNNLERGLG

MSPDBL2 (Gene id: PF3D7_1036300; also referred to as MSP3.8)

Allelic antigens

3D7

FNKCPTEEICKDFSNLPQCRKNVHERNNWLGSSVKNFSSDNKGVLVPPRRQSLCLRITLQDFRTKKKKEGDFEKFIYSYASSEARKLRTIHNNNLEKAHQAIR
YSFADIGNIIRGDDMMDTPTSKETITYLEKVLKIYNENNDKPKDAKKWWTENRHHVWEAMMCGYQSAQKDNQCTGYGNIDDIPQFLRWFREWGTYVC
EESEKNMNTLKAVCFPKQPRTEANPALTVHENEMCSSTLKKYEEWYNKRKTEWTEQSIKYNNDKINYTDIKTLSPSEYLIEKCPECKCTKKN

T9/96

HYKCPDKNFCNGIQNVPNCPLKDFTGTKGDWASSNVRNFLTVNKGVLVPPRRKQMCFRININNFPKLKKTEGKFENFIYSSAGSEAKQLIKLYGNNTEKAL
QAMKYGFADIGNIVQGNDMIDTPTSNKTKTYLEEVLGKQYKNVNDPKDAKTWWIQNKHRVWDAMMCGYQYEKKDNKCTGYGNIYDIPQFLRWFREW
GTYVCEESEKNMNTLKAVCFPKQPRTEANPALTVHENEMCSSTLKKYEEWYNKRKTEWTEQSIKYNNDKINYTDIKTLSPSEYLIEKCPECKCTKKN

Conserved antigens

N-terminal

STCFVVNEGNPNLRNNIINDDELKGKAYNNTIDANNQNIEYNKNLKHNVNSSHISKFSDIMDQEDKGD

C-terminal

SNVGSIQEVNQGSVSEESHSKTIDPSKIDDRLELSSGSSSLEQHSKEDVKKG

SURFIN4.2 (Gene id: PF3D7_0424400)

Allelic antigens

3D7A

RGNALKKISNGDDNYKSPSSNYIEVDCAEDKYFLLEDGTNQSENSCKTKYNYFVSNDYDGTGSAIYSTDQVPSREEIKSPDSLSTLDARGSTHNLNVSNEG

3D7B

RSGSDGRSNIKHIISNNFDDSNMIFPTFNFDFDILKAEEEVLPVNNSDIIYGHEEVEETTQEGASIFEKHSHTSSQQNDSSASGNKYRMLSTTMELPNQQEVFGLYSP

K1A

RGNSLKKISNGDDNYKSPSSNYIEVDCAEDKFLLLEDGTNQSENSCKTIYDYFVSNDYDGTGSAIYSTDQVPRMEEIKSPASLSTLDARGSTHNLNVSNEG

K1B

RSGLDGSSNMAHFISTNFGGNNIFSSTDNLYFDIVRPAREVLPESKSKIIHEHQEMEETTEEGASIIERHSRTSSQNNDAGTSDYMYRILSTTMEFPNEKETFGLYSP

Conserved antigen

DYIFLDEQKEDVCLDEAKNDILSKESFDDVLIKVVDREINREINGKING

Supplementary Figure S1C

```
PF10_0348_3D7       NHNKCPDENFCKGIKNVLSCPPKNSTGRNGDWISVAVKESSTTNKGVLVPPRRTKLCLR- 59
PF10_0348_D6        NHNKCPDENFCKGIKNVLSCPPKNSTGRNGDWISVNVKESSTTNKGVLVPPRRKQMCFRI 60
PF10_0348_Fcc2      NHNKCPDENFCKGIKNVLSCPPKNSTGRNGDWASSNVRNFLTVNKGVLVPPRRKQMCFRI 60
PF10_0348_Palo      NHNKCPDKNFCNGIQNVPNCPLKNFTGTKGDWASSNVRNFLTVNKGVLVPPRRKQMCFRI 60
PF10_0348_7G8       NHNKCPDKNFCNGIQNVPNCPLKDFTGTKGDWASSNVRNFLTVNKGVLVPPRRKQMCFRI 60
PF10_0355_3D7       -FNKCPTEEICKDFSNLPQCRKN--VHERNNWLGSSVKNFSSDNKGVLVPPRRQSLCLRI 57
PF10_0355_T996      -HYKCPDKNFCNGIQNVPNCPLKDFTGTKGDWASSNVRNFLTVNKGVLVPPRRKQMCFRI 59
                      . *** :::*:.:.*: .*  :  .  ..:* .  *::  : ********** .:*:*

PF10_0348_3D7       NINKVWHRIKDEKNFKEEFVKVALGESNALMKHYKEKNLNALTAIKYGFSDMGDIIKGTD 119
PF10_0348_D6        NINNFPKLKKTEGKFENFIYSSAGSEAKQLIKLYGNNTEKAHQAIRYSFADIGNIIRGDD 120
PF10_0348_Fcc2      NINNFPELKKTEGKFENFIYSSAGSEAKQLIKLYGNNTEKALQAMKYGFADIGNIVQGND 120
PF10_0348_Palo      NINNFPELKKTEGKFENFIYSSAGSEAKQLIKLYGNNTEKALQAMKYGFADIGNIVQGND 120
PF10_0348_7G8       NINNFPKLKKTEGKFENFIYSSAGSEAKQLIKLYGNNTEKALQAMKYGFADIGNIVQGND 120
PF10_0355_3D7       TLQDFRTKKKKEGDFEKFIYSYASSEARKLRTIHNNNLEKAHQAIRYSFADIGNIIRGDD 117
PF10_0355_T996      NINNFPKLKKTEGKFENFIYSSAGSEAKQLIKLYGNNTEKALQAMKYGFADIGNIVQGND 119
                    .::..    * * .*:: : . * .*:. * . : ::  :*  *::*.*:*:*:*::* *

PF10_0348_3D7       LIDYQITKNINRALDKILRN-ETSNDKIKKRVDWWEANKSAFWDAFMCGYKVHIGNKPCP 178
PF10_0348_D6        MMDTPTSKETITYLEKVLKIYNENNDKPKDAKKWWTENRHHVWEAMMCGYQSAQKDNQCT 180
PF10_0348_Fcc2      MIDTPTSNKTKTYLEEVLGKQYKNVNDPKDAKTWWIQNKHRVWDAMMCGYKVHIGNKPCP 180
PF10_0348_Palo      MIDTPTSNKTKTYLEEVLGKQYKNVNDPKDAKTWWIQNKHRVWDAMMCGYKVHIGNKPCP 180
PF10_0348_7G8       MIDTPTSNKTKTYLEEVLGKQYKNVNDPKDAKTWWIQNKHRVWDAMMCGYQYEKKDNKCT 180
PF10_0355_3D7       MMDTPTSKETITYLEKVLKIYNENNDKPKDAKKWWTENRHHVWEAMMCGYQSAQKDNQCT 177
PF10_0355_T996      MIDTPTSNKTKTYLEEVLGKQYKNVNDPKDAKTWWIQNKHRVWDAMMCGYQYEKKDNKCT 179
                    ::*   :::    *:::*     . :. *.   **  *:  .*:*:****:    :: *.

PF10_0348_3D7       EHDNMDRIPQYLRWFREWGTYVCSEYKNKFEDVIKLCNIQQFTNQDDSQLLEISKKDKCK 238
PF10_0348_D6        GYGNIDDIPQYLRWFREWGTYVCSEYKNKFEDVIKLCNIQQFTNQDDSQLLEISKEDKCK 240
PF10_0348_Fcc2      EHDNMDRIPQYLRWFREWGTYVCSEYKNKFENVIELCNVRQITNQNDSQLLEISKKDKCK 240
PF10_0348_Palo      EHDNMDRIPQYLRWFREWGTYVCSEYKNKFENVIELCNVRQITNQNDSQLLEISKKDKCK 240
PF10_0348_7G8       GYGNIYDIPQYLRWFREWGTYVCSEYKNKFEDVIKLCNIQQFTNQDDSQLLEISKKDKCK 240
PF10_0355_3D7       GYGNIDDIPQFLRWFREWGTYVCEESEKNMNTLKAVCFPKQPRTEAN-PALTVHENEMCS 236
PF10_0355_T996      GYGNIYDIPQFLRWFREWGTYVCEESEKNMNTLKAVCFPKQPRTEAN-PALTVHENEMCS 238
                     :.*:  ***:************.* ::::: :  :*  :*  .: :   * : ::: *.

PF10_0348_3D7       EALKHYEEWVNRRRPEWKGQCDKFEKEKSKYEDTKSITAEKYLKEICSECDCKYKDLDNT 298
PF10_0348_D6        GALKHYEEWVNRRRPEWKGQCDKFEKEKSKYEDTKSRTAEIYLKQKCSECDCKYKDLDNT 300
PF10_0348_Fcc2      GALKHYEEWVNRRRPEWKGQCDKFEKEKSKYEDTKSRTAEIYLKEICSECDCKYKDLDNT 300
PF10_0348_Palo      GALKHYEEWVNRRRPEWKGQCDKFEKEKSKYEDTKSRTAEIYLKEICSECDCKYKDLDNT 300
PF10_0348_7G8       EALKHYEEWVNRRRPEWKGQCDKFEKEKSKYEDTKSITAEKYLKEICSECDCKYKDLDNT 300
PF10_0355_3D7       STLKKYEEWYNKRKTEWTEQSIKYNNDKINYTDIKTLSPSEYLIEKCPECKCTKKN---- 292
PF10_0355_T996      STLKKYEEWYNKRKTEWTEQSIKYNNDKINYTDIKTLSPSEYLIEKCPECKCTKKN---- 294
                     :**:**** *:*:.**. *. *::::* :* * *: :.. ** : *.**.*. *:

PF10_0348_3D7       FKEF 302
PF10_0348_D6        FKEF 304
PF10_0348_Fcc2      FKEF 304
PF10_0348_Palo      FKEF 304
PF10_0348_7G8       FKEF 304
PF10_0355_3D7       ----
PF10_0355_T996      ----
```

Supplementary Figure S2. Examples of parasites stained in immunofluoresence assay (IFA) with murine sera raised to each of the 16 recombinant antigens. Antibodies in the murine reacted with fixed 3D7 mature stage parasites, and a single serum sample (1/500 dilution) from each group of five mice per antigen was selected for illustration. Antibodies elicited to the MSPDBL1 and SURFIN4.2 recombinant antigens reacted with the majority of mature stage schizonts, whereas the MSPDBL2 antigen was expressed in only a minority of mature stage schizonts (as previously described in detail by Amambua-Ngwa *et al.* with additional IFA data (18)). Antigen specific reactivity is shown in green (Alex Fluor 488) with the nuclei of schizonts in the same field stained with DAPI (Blue).

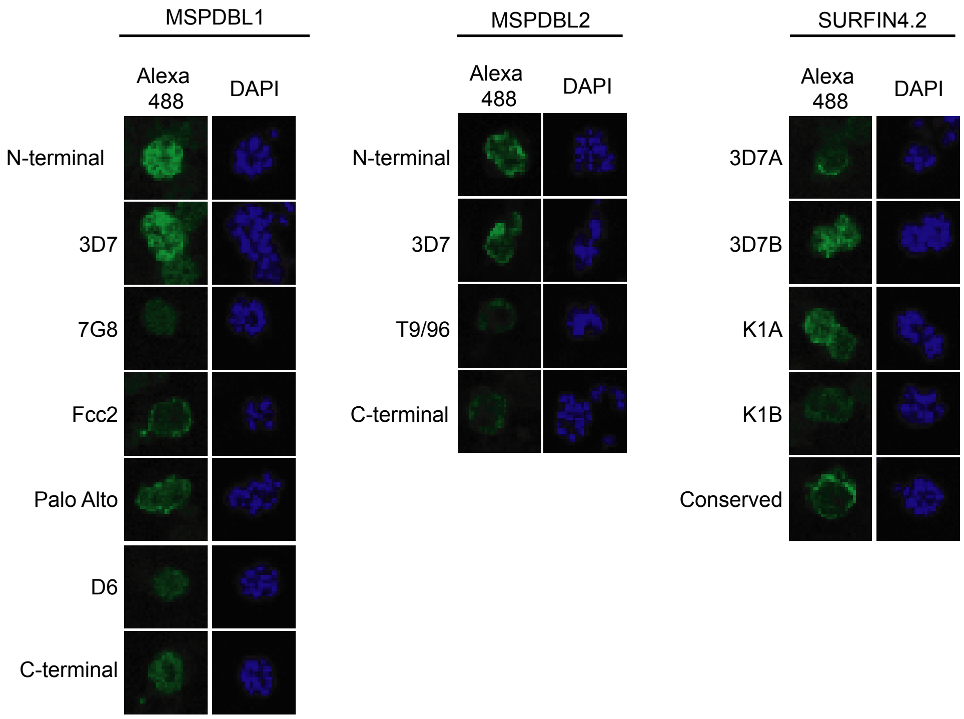
MSPDBL1
Alexa 488
DAPI
N-terminal
3D7
7G8
Fcc2
Palo Alto
D6
C-terminal
MSPDBL2
Alexa 488
DAPI
N-terminal
3D7
T9/96
C-terminal
SURFIN4.2
Alexa 488
DAPI
3D7A
3D7B
K1A
K1B
Conserved

Supplementary Figure S3.

Competition ELISAs to test for the presence of cross-reactive, conformational and linear epitopes in recombinant antigens. Serum samples from Gambian adults (G16 and G31) were used to screen non-denatured and heat-denatured MSPDBL1 and MSPDBL2 antigens, using an approach previously described in the study of other antigens (12). Heat-denatured samples were boiled at 95^0C for 10 min and allowed to cool to room temperature. Non-denatured and heat-denatured proteins were then incubated at a series of diluted amounts with sera for 5 hours at room temperature. Panels A, C, E and G, show that serum reactivity against Palo Alto [MSPDBL1] and T9/96 [MSPDBL2] (serum G16,) and D6 [MSPDBL1] and T9/96 [MSPDBL2] (serum G31) is always fully competed by homologous antigen and partially competed by particular heterologous antigens to differing extents indicating some cross reactivity as well as allele specificity. Panels B, D, F and H, show that heat denaturation of the soluble antigens substantially reduces the competition for antibody binding with the homologous and heterologous coating antigens.

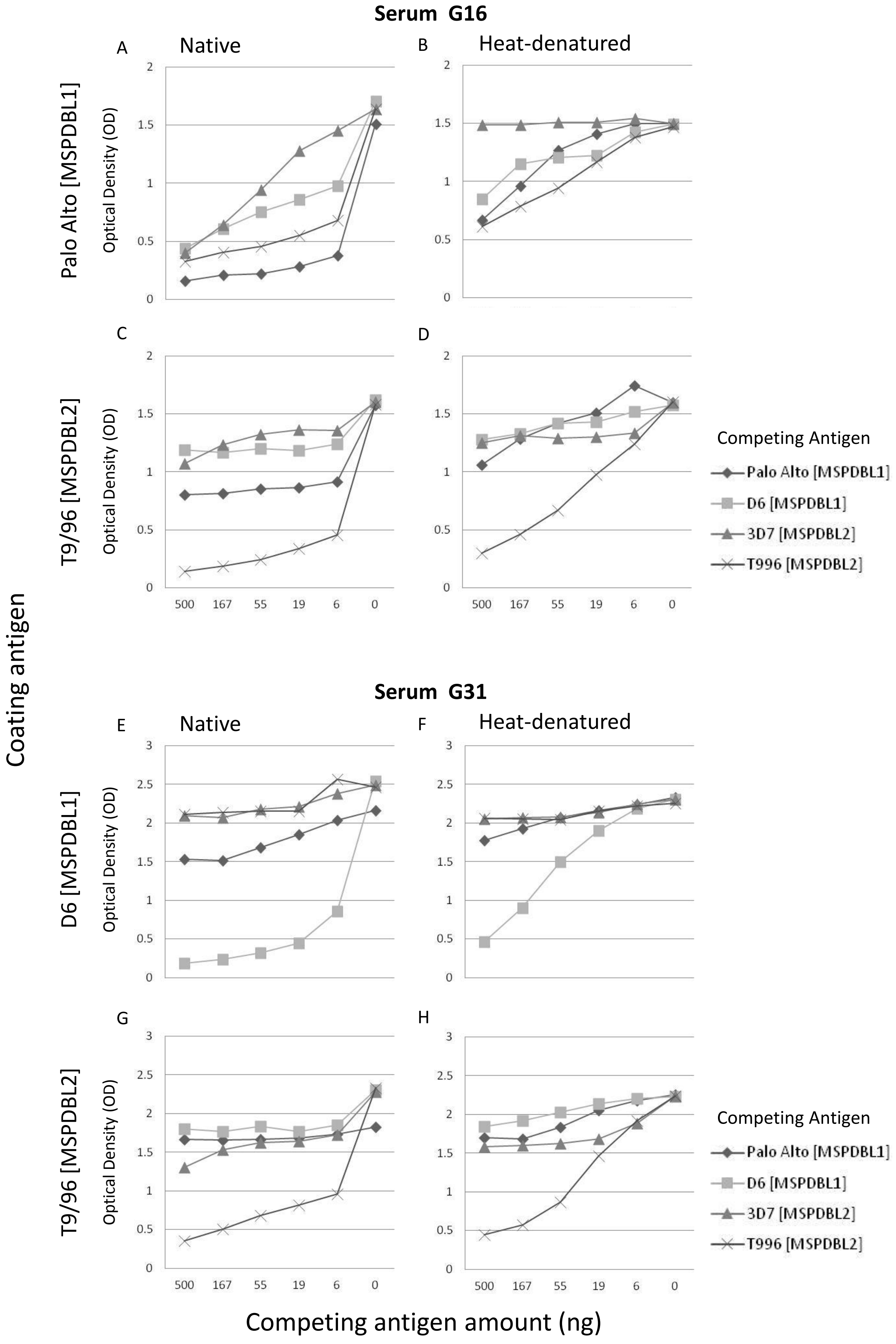

Serum G16
A
Native
B
Heat-denatured
Palo Alto [MSPDBL1]
Optical Density (OD)
C
D
T9/96 [MSPDBL2]
Competing Antigen
Palo Alto [MSPDBL1]
D6 [MSPDBL1]
3D7 [MSPDBL2]
T996 [MSPDBL2]
500
167
55
19
6
0
Coating antigen
Serum G31
E
Native
F
Heat-denatured
D6 [MSPDBL1]
G
H
T9/96 [MSPDBL2]
Competing antigen amount (ng)

Supplementary Table S1 A. Tabulated Pearsons correlates from pairwise analysis for the full panel of 16 antigens for Chonyi. Blue shading highlights Pearson's cross-correlation values (>0.6, P < 0.0001) for within antigen comparisons with the exception of *mspdbl1* and *mspdbl2* , where cross-correlation are between the DBL domain of each antigen.

		PF10_0348							PF10_0355				PFD1160w				
		N-term	C-term	3D7	7G8	Fcc2	Palo Alto	D6	N-term	C-term	3D7	T996	Cons	3D7A	3D7B	K1A	K1B
PF10_0348	N-term	1.00															
	C-term	0.22	1.00														
	3D7	0.41	0.21	1.00													
	7G8	0.46	0.19	0.84	1.00												
	Fcc2	0.40	0.16	0.81	0.91	1.00											
	Palo Alto	0.38	0.12	0.79	0.91	0.96	1.00										
	D6	0.39	0.17	0.81	0.89	0.93	0.88	1.00									
PF10_0355	N-term	0.38	0.20	0.26	0.31	0.31	0.30	0.32	1.00								
	C-term	0.20	0.20	0.20	0.26	0.25	0.24	0.25	0.71	1.00							
	3D7	0.40	0.23	0.61	0.72	0.72	0.71	0.75	0.41	0.28	1.00						
	T996	0.45	0.23	0.73	0.89	0.90	0.93	0.85	0.36	0.26	0.83	1.00					
PFD1160w	Cons	0.18	0.06	0.14	0.16	0.18	0.17	0.17	0.22	0.22	0.20	0.19	1.00				
	3D7A	0.23	0.12	0.18	0.22	0.21	0.19	0.22	0.34	0.18	0.29	0.24	0.15	1.00			
	3D7B	0.30	0.12	0.25	0.27	0.27	0.24	0.26	0.36	0.29	0.31	0.27	0.10	0.30	1.00		
	K1A	0.32	0.18	0.21	0.26	0.24	0.23	0.25	0.36	0.22	0.30	0.29	0.18	0.81	0.29	1.00	
	K1B	0.22	0.15	0.22	0.20	0.18	0.16	0.20	0.13	0.09	0.23	0.18	0.08	0.18	0.39	0.17	1.00

Supplementary Table S1 B. Tabulated Pearsons correlates from pairwise analysis for the full panel of 16 antigens for Ngerenya. Blue shading highlights Pearson's cross-correlation values (>0.6, P < 0.0001) for within antigen comparisons with the exception of *mspdbl1* and *mspdbl2* , where cross-correlation are between the DBL domain of each antigen.

		PF10_0348							PF10_0355				PFD1160w				
		N-term	C-term	3D7	7G8	Fcc2	Palo Alto	D6	N-term	C-term	3D7	T996	Cons	3D7A	3D7B	K1A	K1B
PF10_0348	N-term	1.00															
	C-term	0.07	1.00														
	3D7	0.32	0.12	1.00													
	7G8	0.30	0.15	0.82	1.00												
	Fcc2	0.27	0.10	0.77	0.89	1.00											
	Palo Alto	0.26	0.10	0.75	0.91	0.95	1.00										
	D6	0.21	0.15	0.73	0.79	0.87	0.81	1.00									
PF10_0355	N-term	0.08	0.55	0.13	0.16	0.10	0.12	0.15	1.00								
	C-term	0.03	0.01	0.03	-0.03	-0.03	-0.02	0.01	0.27	1.00							
	3D7	0.21	0.13	0.57	0.66	0.71	0.71	0.65	0.20	0.03	1.00						
	T996	0.31	0.09	0.65	0.85	0.86	0.92	0.70	0.15	-0.01	0.79	1.00					
PFD1160w	Cons	0.15	0.06	0.12	0.15	0.16	0.16	0.17	0.13	0.08	0.14	0.19	1.00				
	3D7A	0.14	0.14	0.17	0.17	0.12	0.14	0.11	0.25	0.11	0.12	0.15	0.21	1.00			
	3D7B	0.09	0.10	0.17	0.14	0.13	0.15	0.12	0.20	0.08	0.09	0.11	0.17	0.70	1.00		
	K1A	0.22	0.12	0.17	0.18	0.15	0.16	0.14	0.17	0.06	0.08	0.14	0.26	0.78	0.70	1.00	
	K1B	0.12	0.14	0.12	0.12	0.08	0.08	0.11	0.26	0.11	0.07	0.08	0.17	0.82	0.78	0.74	1.00

Supplementary Table S2. Association between the presence of serum IgG to the panel of 16 antigens in children aged <11 years and parasite slide positive in October 2000 in Chonyi village and the occurrence of an episode of clinical malaria over the following 6 months

	[a]Proportion of children acquiring malaria who were:				
Antigen	IgG positive	IgG negative	Univariate RR (95% CI))	[b]Multivariate RR (95% CI)	*P-value*
[c] *Chonyi village (<11 years and parasite slide positive n = 119)*					
PF10_348					
N-term	35% (15/43)	33% (25/176)	1.06 (0.63 - 1.79)	1.26 (0.77 - 2.07)	0.358
C-term	36% (5/14)	33% (35/105)	1.07 (0.50 - 2.28)	1.28 (0.55 - 2.91)	0.567
3D7	30% (29/96)	48% (11/23)	0.63 (0.37 - 1.07)	1.31 (0.78 - 2.18)	0.307
7G8	29% (30/104)	67% (10/15)	0.43 (0.27 - 0. 69)	0.84 (0.50 - 1.39)	0.487
Fcc2	30% (33/111)	88% (7/8)	0.34 (0.23 - 0.50)	0.69 (0.43 - 1.13)	0.144
Palo Alto	29% (31/106)	69% (9/13)	0.42 (0.26 - 0.68)	0.83 (0.52 - 1.32)	0.429
D6	30% (32/108)	73% (8/11)	0.41 (0.26 - 0.65)	0.75 (0.49 - 1.15)	0.186
PF10_0355					
N-term	18% (3/17)	36% (37/102)	0.49 (0.17 - 1.41)	0.73 (0.29 - 1.82)	0.500
C-term	26% (8/31)	36% (32/88)	0.71 (0.37 - 1.37)	1.04 (0.56 - 1.92)	0.899
3D7	23% (22/97)	82% (18/22)	0.28 (0.18 - 0.42)	0.51 (0.28 - 0.93)	**0.029***
T996	31% (36/115)	100% (4/4)	0.31 (0.24 - 0.41)	0.61 (0.35 - 1.05)	0.075
PFD1160w					
C-term	30% (16/53)	36% (24/66)	0.83 (0.49 - 1.40)	1.02 (0.63 - 1.66)	0.933
3D7A	6% (1/17)	38% (39/102)	0.15 (0.02 - 1.05)	0.25 (0.04 - 1.73)	0.159
3D7B	17% (4/23)	38% (36/96)	0.46 (0.18 - 1.18)	0.76 (0.31 - 1.86)	0.552
K1A	29% (9/31)	35% (31/88)	0.82 (0.44 - 1.53)	1.12 (0.61 - 2.05)	0.721
K1B	42% (5/12)	33% (35/107)	1.27 (0.62 - 2.63)	1.32 (0.70 - 2.46)	0.391

RR, Risk Ratio; CI, Confidence Interval

[a] Number of individuals developing malaria/the total number of individuals that were IgG positive or negative. [b] The incidence Risk Ratio was estimated from multivariate analysis after adjusting for age and reactivity to Plasmodium falciparum schizont extract in generalized linear models. [c] Analysis focused on individuals who were parasitaemic at the time of serum sampling in October 2000.

Supplementary Table S3. Association between the presence of serum IgG to the panel of 16 antigens in children aged <11 years and parasite slide positive in October 2000 in Ngerenya village and the occurrence of an episode of clinical malaria over the following 6 months

	[a]Proportion of children acquiring malaria who were:				
Antigen	IgG positive	IgG negative	Univariate RR (95% CI)	[b]Multivariate RR (95% CI)	*P-value*
[c]Ngerenya village (<11 years and parasite slide positive n = 61)					
PF10_348					
N-term	29% (2/7)	48% (26/54)	0.59 (0.18 - 2.00)	0.63 (0.25 - 1.59)	0.326
C-term	0% (0/1)	47% (28/60)	nd	nd	nd
3D7	38% (11/29)	53% (17/32)	0.71 (0.40 - 1.27)	0.82 (0.48 - 1.41)	0.480
7G8	28% (7/25)	58% (21/36)	0.48 (0.24 - 0.96)	0.55 (0.27 - 1.12)	0.100
Fcc2	39% (12/31)	53% (16/30)	0.73 (0.41 - 1.27)	0.84 (0.51 - 1.40)	0.510
Palo Alto	32% (12/38)	70% (16/23)	0.45 (0.26 - 0.78)	0.53 (0.32 - 0.89)	**0.017***
D6	35% (7/20)	51% (21/41)	0.68 (0.35 - 1.34)	0.76 (0.39 - 1.47)	0.409
PF10_0355					
N-term	50% (1/2)	46% (27/59)	1.09 (0.26 - 4.54)	0.93 (0.48 - 1.81)	0.831
C-term	42% (11/26)	49% (17/35)	0.87 (0.49 - 1.54)	1.06 (0.63 - 1.79)	0.825
3D7	24% (8/33)	71% (20/28)	0.34 (0.18 - 0.65)	0.38 (0.18 - 0.82)	**0.014***
T996	36% (12/33)	57% (16/28)	0.64 (0.36 - 1.11)	0.85 (0.48 - 1.51)	0.578
PFD1160w					
C-Term	45% (5/11)	46% (23/50)	0.99 (0.48 - 2.03)	0.85 (0.47 - 1.53)	0.582
3D7A	33% (2/6)	47% (26/55)	0.71 (0.22 - 2.83)	0.77 (0.33 - 1.80)	0.539
3D7B	25% (1/4)	47% (27/57)	0.53 (0.09 - 2.99)	0.54 (0.14 - 2.05)	0.367
K1A	33% (2/6)	47% (26/55)	0.71 (0.22 - 2.28)	0.76 (0.32 - 1.80)	0.534
K1B	33% (2/6)	47% (26/55)	0.71 (0.22 - 2.28)	0.77 (0.33 - 1.80)	0.545

RR, Risk Ratio; CI, Confidence Interval; nd, not netermined (insufficient datapoints to calculate)

[a] Number of individuals developing malaria/the total number of individuals that were IgG positive or negative. [b] The incidence Risk Ratio was estimated from multivariate analysis after adjusting for age and reactivity to *Plasmodium falciparum* schizont extract in generalized linear models. [c] Analysis focused on individuals who were parasitaemic at the time of serum sampling in October 2000.
